# Competition alters species’ plastic and genetic response to environmental change

**DOI:** 10.1038/s41598-021-02841-8

**Published:** 2021-12-07

**Authors:** Lynn Govaert, Luis J. Gilarranz, Florian Altermatt

**Affiliations:** 1 Department of Evolutionary Biology and Environmental Studies, University of Zurich, Winterthurerstrasse 190, 8057 Zurich, Switzerland; 2 Department of Aquatic Ecology, Eawag: Swiss Federal Institute of Aquatic Science and Technology, Überlandstrasse 133, 8600 Dübendorf, Switzerland; 3 URPP Global Change and Biodiversity, University of Zurich, Winterthurerstrasse 190, 8057 Zurich, Switzerland; 4 Present Address: Leibniz-Institute of Freshwater Ecology and Inland Fisheries (IGB), Müggelseedamm 310, 12587 Berlin, Germany

**Keywords:** Community ecology, Evolutionary ecology, Experimental evolution

## Abstract

Species react to environmental change via plastic and evolutionary responses. While both of them determine species’ survival, most studies quantify these responses individually. As species occur in communities, competing species may further influence their respective response to environmental change. Yet, how environmental change and competing species combined shape plastic and genetic responses to environmental change remains unclear. Quantifying how competition alters plastic and genetic responses of species to environmental change requires a trait-based, community and evolutionary ecological approach. We exposed unicellular aquatic organisms to long-term selection of increasing salinity—representing a common and relevant environmental change. We assessed plastic and genetic contributions to phenotypic change in biomass, cell shape, and dispersal ability along increasing levels of salinity in the presence and absence of competition. Trait changes in response to salinity were mainly due to mean trait evolution, and differed whether species evolved in the presence or absence of competition. Our results show that species’ evolutionary and plastic responses to environmental change depended both on competition and the magnitude of environmental change, ultimately determining species persistence. Our results suggest that understanding plastic and genetic responses to environmental change within a community will improve predictions of species’ persistence to environmental change.

## Introduction

The rate of anthropogenically-induced environmental change is nowadays faster than ever^1^, including changes in temperature and precipitation^2^, pollution by nutrients^3^, salts^4^, and synthetic chemicals^5^. Such rapid changes in abiotic factors have resulted in losses of biodiversity and associated services^6,7^. Previous studies have shown how individual species have the ability to phenotypically track environmental changes or to migrate to novel habitats in order to survive^8,9^. Phenotypic tracking of environmental conditions occurs either via phenotypic plasticity or via evolution. Phenotypic plasticity is the capacity of a single genotype to produce a range of phenotypes under varying environments^10^. It is a within-generation individual response mechanism that can occur rapidly to enable short-term survival of organisms to changing environments^11,12^. Evolution is the genetic adaptation through mutations or via the selection of existing genotypes^13^. Evolution occurs across generations and therefore is often a slower response to environmental change, nevertheless, crucial for long-term survival of species^11,12^.

Predicting species’ responses to environmental change requires partitioning phenotypic responses into its plastic and genetic components^14,15^. Yet, species’ plastic and genetic responses to environmental change are mostly studied in isolation of each other^12,16^, in a single-species perspective^17,18^, or in simple environmental contrasts (i.e. a control versus a single treatment condition only^19^). It is expected that the direction and magnitude of plasticity and evolutionary trait change would differ depending on the magnitude of environmental change, and whether selection occurred within a multi-species setting such as a community as opposed to a single species^15^.

In virtually all natural systems, organisms of different species are embedded in communities, in which direct and indirect ecological effects among species can also exert selection pressures on each member of the community^20,21^, for example through competition for limiting resources^22^ or space^19^. Previous studies have shown that species interactions (such as competition, predation, or parasitism) can influence species’ responses to environmental change^19,22–24^ and that this effect can even depend on the abiotic environment^21^. These studies have shown that species respond differently to a novel environment depending whether they occur alone or in the presence of other species^22^. This, because the presence of other species most likely changes the fitness landscape in which they evolve^19^. Species interactions have also been found to induce phenotypic plasticity as well as evolutionary responses in the focal species (e.g. predator-induced defenses^25^ or competition^26^). More recently, Grainger et al.^27^ showed that interspecific competition can even have legacy effects further determining species’ evolutionary responses to environmental change. To our knowledge, no study has explicitly quantified the relative extent to which plasticity and evolutionary changes contribute to the observed phenotypic responses to environmental change when species are embedded within a community. Therefore, it remains largely unknown whether species interactions, such as interspecific competition, can alter the plastic and genetic response to the abiotic change, whether the effect of interspecific competition depends on the magnitude of the abiotic change, and whether this effect varies among different member species of the community.

Here, we used three naturally co-occurring and competing freshwater ciliate species^28^ (*Paramecium aurelia*, *Spirostomum teres,* and *Tetrahymena thermophila*) to experimentally quantify plastic and genetic responses to abiotic environmental change (here: salinity) within the absence and presence of competing species. We use freshwater ciliates as model organisms because they are easy to maintain and manipulate in the laboratory, and they have short generation times. There also exists standardized protocols and automated methods for quantification and phenotyping of these organisms, which allows addressing a wide range of ecological and evolutionary questions^29^. Moreover, freshwater ciliates and other protists are a major part of the global biomass^30^, and play a key role in the aquatic food web by providing nutrition for higher trophic levels^31^, and by being important grazers of bacteria^32^. The species used are known to be naturally competing for similar resources, and have a life history and ecology that makes them suitable for such comparisons (see also^29^). The use of salt as a stressor reflects the increasing global problem of salinization of freshwater systems^33,34^. It is expected that its devastating effects on freshwater systems will intensify with increasing climate change^35^.

We conducted an experiment in two phases. First, we performed a long-term selection experiment exposing replicated populations of each species or replicated communities of all three species to an environmental gradient of increasing salinity (five levels). This resulted into 20 unique experimental treatment combinations (i.e. 5 salinity environments × 4 species treatments; each replicated 3 times), run for 78 days (corresponding to at least 50–150 generations). From the selection phase we can determine whether and how interspecific competition alters phenotypic trajectories to abiotic environmental change varying in magnitude.

**Figure 1:**
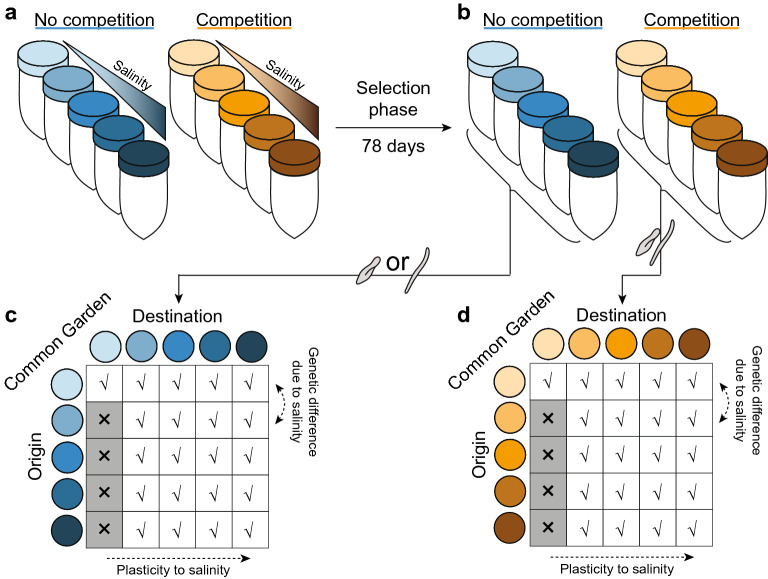
Experimental set-up. (**a**) During the selection phase, populations of all species were kept in five different environmental conditions (represented by color shades) either in absence (blue colors) or presence (orange colors) of competing species (each threefold replicated). (**b**) End of the selection phase. Change in trait means from (**a**) to (**b**) reflect the phenotypic temporal response to salinity and competition. (**c**,**d**) Common garden experiment in which aliquots of the populations of all species evolved in the absence (**c**) and presence (**d**) of competition are placed in a common salinity environment, respectively. In this study, for the competition treatment an aliquot of the end point of the selection phase was used in the common garden. Comparing trait means between (**c**) and (**d**) shows whether the response to salinity depends on the competition and salinity condition the species was selected in.

Then, to disentangle plastic and genetic components of observed trait changes, the populations and communities at the end of the selection phase (further referred to as the selected populations) were used in a common garden experiment (Fig. 1). In this common garden experiment, each selected population or community was exposed to each of the different salt concentrations used in the selection phase (except for the zero salinity selected populations due to the impossibility of transferring individuals without some of the salty media they are embedded in). From the common garden experiment, we can assess (1) whether abiotic environmental change varying in magnitude results in different plastic and genetic responses of the populations evolved without competition and (2) whether these responses are altered by competition. By using the entire community in a common garden (versus singling out the individual species), we can evaluate whether the presence of competing species can mask plastic or genetic responses to abiotic environmental change. When competition between species is weak, plastic and genetic responses to abiotic environmental change in the absence and presence of competition would be comparable^17^. However, in cases of strong competition, we might expect plastic and genetic responses to environmental change to be accelerated or inhibited when competition selects or does not select for similar trait values in the new environment^36^.

As multiple species were used, we can additionally assess whether patterns found vary among species. Consequently, we can understand when interspecific competition, and more generally the community context, is critical to determine plastic and evolutionary trajectories of species, a question currently remaining largely unclear^37^. Furthermore, our study allows understanding species’ responses to environmental change. It also extends the partitioning of plastic and genetic components towards a multi-species perspective, a crucially needed step to predict species responses to environmental change^38,39^.

We quantify species’ responses by measuring key traits of all species at the scale of individuals, using highly-resolved and automated video analyses (detailed in “Methods”). The measured traits—biomass (i.e. the area of a cell), cell shape (i.e. ratio of the largest to second largest cell size axis of an individual^40^), and dispersal ability (i.e. gross speed)—are directly linked to ecological, physiological, and behavioral responses, as well as fitness of these species under environmental change^29,41^. Based on previous studies^42–44^, we expect rapid plastic responses towards lower biomass, cell shape, and dispersal ability. We expect larger contributions of mean trait evolution in the higher salt concentrations as these conditions might express stronger selection. However, competing species may select for different trait values^45^, and may therefore amplify or impede trait responses to salinity. If a species’ plastic and genetic response to competing species are opposite to its plastic and genetic response to the abiotic environmental change, then initially one might not observe phenotypic change. Only when disentangling the trait change into plastic and genetic components, effects of the abiotic and biotic (here: presence of competing species) environment would be detected.

## Results

### Phenotypic response to salinity and presence of competing species during the selection phase

**Figure 2:**
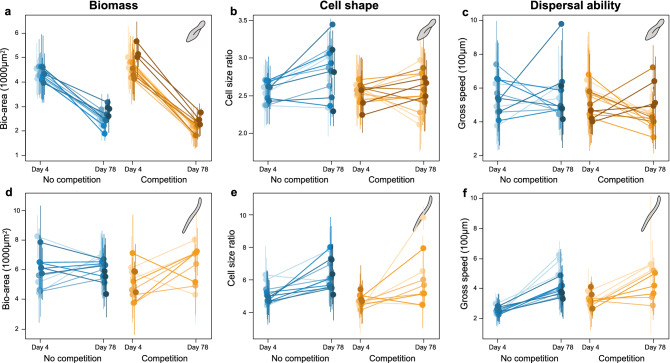
Temporal phenotypic response of *Paramecium aurelia* (**a**–**c**) and *Spirostomum teres* (**d**–**f**) during the selection phase. Trait values at the start (day 4) and end (day 78) of the selection phase for biomass, quantified as bio-area (**a**,**d**), cell shape, quantified as cell size ratio between the major and minor cell axis (**b**,**e**), and dispersal ability, quantified as gross speed (**c**,**f**) when *P. aurelia* and *S. teres* evolved in the absence (blue colors) and presence (orange colors) of competing species along different salinity conditions (indicated by different shades, from light to dark color intensity corresponding to 0, 0.5, 1, 2 and 4 g/l). Dots represent mean trait values of each replicate with error bars showing the standard deviation and representing the phenotypic trait distribution. The slope of the line connecting dots shows the temporal shift in the trait considered. Dots are horizontally jittered for visual aid. Temporal phenotypic differences are shown in Supplementary Figure S1. Supplementary Tables S1 and S2 show the detailed results of the statistical analysis.

Across all replicates, *P. aurelia* and *S. teres* changed phenotypically over the duration of the selection experiment (Fig. 2). The third species, *T. thermophila*, drastically declined in population density throughout all replicates. In the absence of competition, this density reduction occurred around day 25 for the lowest salinity conditions and around day 20 for the highest salinity conditions. Only for the lowest salinity conditions, low population sizes were maintained throughout the selection phase. In the presence of competition, this density reduction occurred around day 15 for the low salinity conditions and around day 10 for the high salinity conditions. As we did not detect any *T. thermophila* individuals by the end of the selection phase in the presence of competitors, we assumed this species went extinct in these conditions. Due to the lack of trait data for *T. thermophila*, we here focus on results found for *P. aurelia* and *S. teres*. Interestingly, in the two highest salinity conditions (2 and 4 g/L), *S. teres* also went extinct (i.e. its population size fell below the detection threshold) in all replicates, except one of the competition treatment in the 4 g/L salt environment. During the selection phase, *P. aurelia* showed, independent of treatment, a significant reduction in biomass and dispersal ability and a significant increase in cell shape, i.e., individuals became more elongated (Fig. 2a–c). The second species, *S. teres*, showed a significant increase in biomass, cell shape and dispersal ability (Fig. 2d–f). Importantly, for both species, these generally-observed trait changes were significantly altered by competition and the salinity environment. This was detected by a significant statistical interaction between time and interspecific competition, and between time and salinity (Supplementary Tables S1, S2).

Specifically, for *P. aurelia*, the decrease in biomass and dispersal ability was larger for those individuals from the competition treatment (Supplementary Fig. S1). For dispersal ability and cell shape, the effect of competition differed between salinity environments. In the lowest salinity, *P. aurelia* individuals on average decreased in cell shape during the selection experiment (Fig. 2b). Contrary, *P. aurelia* individuals in the highest salinity showed an increase in cell shape—individuals became more elongated. For *S. teres*, individuals from the competition treatment decreased less in biomass on average, but showed a larger increase in cell shape (Supplementary Table S2). However, both of these effects depended on salinity (Supplementary Fig. S1). Competing species had no effect on the temporal response in dispersal ability for *S. teres* (Supplementary Table S2). Thus, at the end of the selection experiment, the abiotic environmental change and the presence of competing species resulted in a set of different trait values compared to those of the ancestral population.

### Phenotypic response to salinity and competing species assessed in the common garden

From the common garden experiment, we evaluated the effect of exposure to the abiotic salinity environment (historical salinity) and to competing species (historical competition) during the selection phase on the trait values of *P. aurelia* and *S. teres*, and whether these effects varied with salinity used in the common garden experiment. Here, we focus on total phenotypic change rather than partitioning this change into its plastic and genetic components (but see next section).

**Figure 3:**
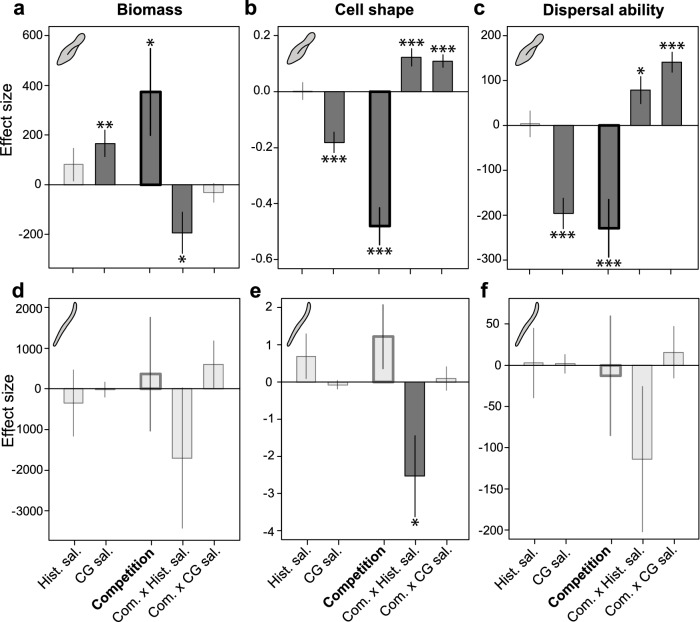
Factors affecting the phenotypic response to salinity. Effect sizes (difference in trait mean) and their standard errors for *Paramecium aurelia* (**a**,**c**)*,* and *Spirostomum teres* (**d**,**f**) obtained from a linear mixed effect model comparing traits of the species evolved in the absence and presence of competition using the common garden data (see “Methods”). Variables displayed are the salinity in which the species evolved in (Historical salinity; Hist. sal.), the salinity a species experienced during the common garden experiment (Common garden salinity; CG sal.), whether the species was evolved in the absence or presence of competing species (Competition), and interaction of competition with historical salinity and common garden salinity (Com. × Hist. sal. and Com. × CG sal., respectively). Measured traits were biomass, quantified as bio-area (**a**,**d**), cell shape, quantified as cell size ratio between the major and minor cell axis (**b**,**e**), and dispersal ability, quantified as gross speed (**c**,**f**). The competition effect is highlighted with bold bars. Significant effects are shown with dark grey bars and asterisks indicating the level of significance, with *p < 0.05, **p < 0.01, ***p < 0.001. Supplementary Figs. 3–4 show the robustness analysis of each of these effect sizes and p values. Supplementary tables S3 and S4 show the detailed results of the statistical analysis.

Overall, we found strong effects of the historical salinity and historical competition, and of the common garden salinity environments on *P. aurelia* traits, but not on *S. teres* traits (Fig. 3). The results of the statistical analysis are given in Supplementary Tables S3 and S4. Historical exposure to competing species had the largest impact on measured traits of *P. aurelia* (Fig. 3a–c). Specifically, competition during the selection phase resulted in larger *P. aurelia* individuals that had a lower cell shape and dispersal ability (Supplementary Fig. S2). However, the effect of historical competition depended on the historical salinity environment (significant interaction between historical competition and historical salinity; Supplementary Table S3). All three traits also showed a significant effect of salinity used during the common garden (Fig. 3a–c). Only for cell shape and dispersal ability, historical competition also altered the response of *P. aurelia* to salinity used in the common garden experiment (significant interaction between the historical competition and salinity environment of the common garden; Supplementary Table S3). Specifically, *P. aurelia* individuals were found to be larger, more spherical and had lower dispersal ability in the higher salinity used in the common garden. The second species, *S. teres*, showed only a marginal response to historical salinity and historical competition (Fig. 3d–f; Supplementary Table S4). Only *S. teres*’ cell shape showed a significant difference in response to historical salinity between the absence and presence of competing species (Fig. 3e). Yet, our robustness analysis shows that such effect was marginal (Supplementary Figs. S3, S4).

### Contribution of phenotypic plasticity and evolution to trait change

In order to predict long-term species persistence, we need to know if species can evolve in response to novel abiotic environmental conditions, and if this evolutionary response differs depending on the strength of the abiotic environmental change and when species evolve in the presence of competing species. To answer this question, we (1) partition trait change from the ancestral–population at the start of the selection phase–to the selected populations into plastic and genetic contributions, (2) quantify phenotypic plasticity to salinity of the different selected populations and (3) compare plastic and genetic differences between selected populations differing in historical competition but experienced the same historical salinity.

To disentangle plastic and genetic responses to salinity, we used the reaction norm approach^15,46^. This method quantifies contributions of plasticity, mean trait evolution, and evolution of plasticity to observed trait changes from the ancestral population to the selected populations. To evaluate whether the effect of competition could alter plastic and genetic responses to environmental change, we similarly used the reaction norm approach from the ancestral population to each of the populations that evolved in the presence of competing species. Trait values obtained from those populations confound a plasticity response to competition, and thus do not reflect accurate genetic changes. Thus, this allows to evaluate whether competition can significantly alter evolution to abiotic environmental change either by inhibiting or augmenting such evolutionary change via a plastic and/or evolutionary response to competition. For this analysis, we need to take into account that *S. teres* went extinct in all except one replicate of the two highest salinity conditions (2 and 4 g/L). Therefore, for these two conditions, the component ‘mean trait evolution’ does reflect genetic change. Moreover, the extinction of *S. teres* allows to further evaluate whether the presence of competing species resulted in a different plastic and evolutionary response of *P. aurelia* to salinity for the two highest salinity conditions.

**Figure 4:**
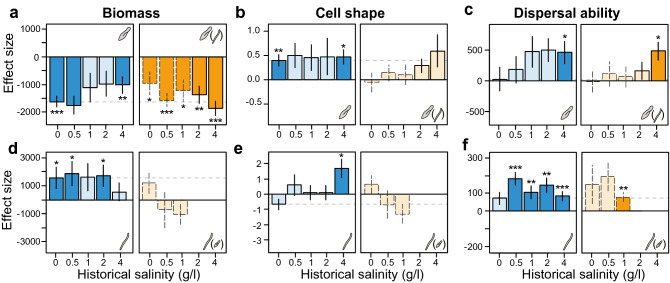
Genetic contributions to phenotypic change along the different salinity conditions and in function of competition. Bars display the magnitude (i.e. effect size) of the genetic responses to salinity and their standard errors for *Paramecium aurelia* (**a–c**) and *Spirostomum teres* (**d–f**) evolved in the absence (blue colors) and presence (orange colors) of competing species for (**a**,**d**) biomass, quantified as bio-area, (**b**,**e**) cell shape, quantified as cell size ratio between the major and minor cell axis, and (**c**,**f**) dispersal ability, quantified as gross speed. For the competition treatment, only the *P. aurelia* populations evolved in the two highest salinity conditions reflect accurate estimates for genetic trait change in this analysis. In the lowest salinity conditions this component is confounded with a plasticity response to the presence of competing species (indicated by dashed bars). Darker colors indicate significant effects, with asterisks referring to the level of significance; *< 0.05, **< 0.01, ***< 0.001. Detailed partitioning into (ancestral) plasticity, mean trait evolution and evolution of plasticity can be found in Supplementary Figs. S5–S6. Summary of statistical results can be found in Tables S7and S8.

For both species in the absence of competition, mean trait evolution showed the largest effect to the observed trait changes (Supplementary Figs. S5, S6) and mainly acted in opposite direction of evolution of plasticity (Supplementary Figs. S5, S6). The ancestral population of *P. aurelia* showed limited plasticity responses to salinity for all three traits (Supplementary Fig. S5). However, the selected populations did show significant phenotypic plasticity in response to salinity for the three traits. Those populations evolved in higher salinity showed the largest plastic responses (Supplementary Fig. S7). For *P. aurelia*, the previously described phenotypic trait change toward lower biomass, higher cell shape and higher dispersal ability can thus be mainly attributed to mean trait evolution (Fig. 4a–c). For biomass, the evolutionary change toward smaller biomass was similar for the selected populations evolved in the two lowest salinity conditions (0 and 0.5 g/L). The selected populations evolved in the higher salinity conditions showed a smaller evolutionary change, and thus evolved larger biomass compared to the populations evolved in low salinity conditions (blue bars in Fig. 4a–c).

The ancestral population of *S. teres* showed limited phenotypic plasticity to salinity for two of the three traits (Supplementary Fig. S8). Only for biomass, a positive plasticity response to increasing salinity was found (Supplementary Fig. S2; Supplementary Table S11). All five selected populations of *S. teres* evolved without competitors showed strong effects of mean trait evolution towards an increase in biomass and dispersal ability (Fig. 4d–f). The effect of mean trait evolution for cell shape was only significant for the highest salinity.

### Competition alters evolution

For *P. aurelia,* the presence of competing species in the common garden assessment altered the effect sizes of mean trait change for the three traits quantified (comparing blue with orange bars in Fig. 4a–c): for biomass, the presence of competitors reduced the effect size between the populations of the zero salinity conditions by half. This strong effect of competition is possibly due to the lack of environmental change in the low salinity condition. For the intermediate salinity conditions, the effect of competition for biomass was weaker. Also, while mean trait change towards an increase in cell shape and dispersal ability was found, this effect disappeared in the presence of competitors for low and intermediate salinity conditions (Fig. 4b,c).

The strong reduction in effect size for these traits in the presence of competitors could indicate that the plastic or evolutionary response to competition could act in opposite direction as the evolutionary response to salinity, masking the genetic response to salinity in these populations. This finding is corroborated by the opposing plasticity response found in the absence and presence of competing species for the selected populations from the zero salinity (Supplementary Fig. S7). For the two highest salinity conditions, effect sizes reflect evolutionary change. We observe a larger effect size of evolutionary trait change toward smaller biomass when *P. aurelia* evolved with competitors compared to when it evolved without competitors. This suggests that competition, even when competitors go extinct, can still influence the evolutionary response to environmental change.

**Figure 5:**
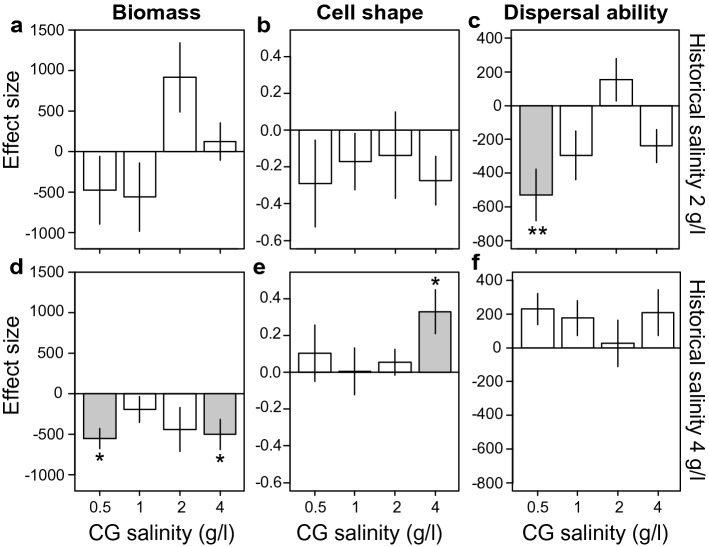
Genetic trait difference between *Paramecium aurelia* selected populations evolved with and without competitors of the two highest salinity conditions. Bars display the magnitude (i.e. effect size) of the genetic trait difference along the different common garden (CG) salinity environments for the *P. aurelia* selected populations for (**a**,**d**) biomass (quantified as bio-area), (**b**,**e**) cell shape (quantified as cell size ratio of the major and minor cell axis) and (**c**,**f**) dispersal ability (quantified as gross speed). Top and bottom row display results for *P. aurelia* populations evolved in the 2 and 4 g/l salinity, respectively (given by historical salinity). Error bars reflect standard errors of the effect size as obtained from the regression model. Grey bars indicate significant effects, with asterisks referring to the level of significance; *< 0.05, **< 0.01, ***< 0.001. Summary of statistical results can be found in Supplementary Table S13. Results of the effect sizes including microcosm ID 120 can be found in Supplementary Figure S12.

Moreover, the evolutionary change towards smaller biomass for *P. aurelia*, when it evolved with competitors compared to when it evolved without competitors, was found to be significant for the selected populations of the highest salinity condition when measured in the low or high salinity common garden environment (Fig. 5d). For the selected populations of *S. teres* evolved with competitors, most effects of mean trait change were opposite to the ones found in the absence of competing species (Fig. 4d–f).

## Discussion

To ensure long-term survival of species, phenotypic responses–both plastic and genetic–to changing abiotic environments have shown to be crucial^47,48^. Yet, many studies evaluate how these responses operate in isolation^12,16^ and within a single-species perspective^17,18^. This ignores a universal feature of all biological systems, namely that species live in communities. Even though an increasing number of studies show the importance of competing species for evolutionary responses to environmental change^27,37,47,48,49^, it remains largely unknown whether interspecific competition can alter the plastic and genetic response to abiotic environmental change, whether the effect of competition depends on the magnitude of the abiotic environmental change, and whether this effect varies among different member species of the community. Quantifying such plastic and genetic responses in relation to the strength of abiotic change in the presence of competitors may be key to understand to what extent species can adapt and persist in a changing world.

Our results contribute to understand how competition modulates plastic and evolutionary trajectories in response to abiotic environmental change. In addition to previous research on species’ responses within multi-species settings^19,21,37^, our results suggest that species persistence within the community is mediated by plastic and genetic responses (or lack thereof) to the abiotic environmental change as well as to competition. While we started the experiment with three species, in the end we could only evaluate plastic and genetic responses of two species. We found that species varied in their plastic and genetic responses to the joint effects of abiotic change and competition with consequences for their persistence. We found that plastic and genetic responses depended on the strength of the abiotic change. More extreme environments often result in rapid evolution^50^. Here too, we found that the higher salinity resulted in larger plastic and genetic responses. However, these responses were altered by competition. More importantly, we found that genetic trait change in response to the abiotic environment can be masked by the plastic response to the presence of competitors. Our study aligns with previous studies, indicating that a single-species approach is insufficient to predict evolution in multi-species scenarios, especially in those where species interactions are expected to be strong^17^. In addition, our study also shows that disentangling plastic and genetic responses of species to environmental change and to competition reveal that these responses may often go in opposite direction resulting in smaller phenotypic changes than expected from a single-species observation.

While the ancestral populations of *P. aurelia* and *S. teres* did not show a strong plastic response against increased salinity and competition initially, the selected populations did show (evolved) plasticity and genetic responses to salinity and competition. This, in some cases, resulted in very different trait values from those of the ancestral populations, in particular for the biomass of *P. aurelia*. While there was a general genetic decrease in biomass observed in the zero salinity environment in the absence of interspecific competition, *P. aurelia* individuals evolved larger biomasses in the highest salinity, but lower biomasses in the same salinity when competing species were present. This reduction in biomass in the presence of competing species has previously been found by terHorst^51^ for *Colpoda* populations. This might indicate that the presence of competing species selects for a different trait optimum and this most likely by altering the fitness landscape in which species evolve^19^.

Our study is also in line with a recent finding by Grainger et al.^27^, demonstrating that competition can have a legacy effect on future evolutionary responses to abiotic environmental change. This legacy effect of competition also had important implications for the evolution of *P. aurelia* to the abiotic environmental change. In the competition treatment, two of its competitors (*T. thermophila* and *S. teres*) went extinct throughout the selection experiment (for *S. teres* this extinction occurred only in the two highest salinity conditions towards the end of the selection phase). Nevertheless, we showed strong differences in trait values for *P. aurelia* populations in the two highest salinity conditions between those populations that evolved with and without competitors. Hence, it is likely that past competition still impacted the evolution of *P. aurelia* to the abiotic environmental change. In addition, we showed that competition can also alter the phenotypic plasticity response to the abiotic environment, and that both evolutionary and plasticity responses depend on the magnitude of the abiotic environmental change.

Two of the three species faced extinction during our experiment. The extinction of *T. thermophila* in most of the conditions was most likely a combination of resource competition^52^ and sensitivity to high salt concentrations. The extinction of *S. teres* at high salinity when grown with competitors, but not without competitors could be due to several reasons. First, compared to *P. aurelia*, *S. teres* only showed limited phenotypic responses to salinity and to competition. Such limited responses may indicate the incapability of *S. teres* to appropriately respond to the changing environment. Second, *S. teres* individuals in high salinity evolved lower biomass values and thus similar cell sizes as *P. aurelia* individuals in the absence of competition (Supplementary Fig. S10). These trait changes increase the niche overlap of these species and could increase competitive strength, which could have contributed to the extinction of *S. teres* in the high salinity in the presence of competition. Last, in the zero-salt concentration, *P. aurelia* biomass showed a negative plasticity response to salinity in the competition treatment, which was opposite to the response in the absence of interspecific competition (Supplementary Fig. S7). As this population only experienced competition and no change in the salinity environment, such opposing plasticity response may indicate a response to competition rather than to salinity. A response to competition of *P. aurelia* was also observed in the genetic trait change, where *P. aurelia* populations increased in biomass in response to higher salinity, but showed a steep decrease in biomass when it evolved in the presence of competing species, potentially showing an adaptive change to competition. For *S. teres*, such responses to competition were not found (Supplementary Fig. S8). The plasticity response to salinity for *S. teres* with competing species resulted in larger individuals, whereas the genetic trait change of *S. teres* resulted in smaller individuals. This opposing response of plasticity and genetic trait change may also explain why *S. teres* went extinct in the higher salinity with competitors.

In line with previous studies, we show that interspecific competition may change the direction of selection^53^. In addition, our results indicate that responses to competition can mask evolutionary change to the abiotic environment if the plasticity response to competition is in the opposite direction as the evolutionary response to the abiotic environmental change. This seems to be the case for two of our traits—cell shape and dispersal ability—for *P. aurelia*. While we did not explicitly evaluate phenotypic plasticity of the selected populations to competition, this would be a worthwhile extension for future studies. A common garden experiment could be designed so that it quantifies plasticity against one or multiple competing species^18^. Species’ responses from the competition treatment could also be evaluated separately for each species in the common garden experiment to estimate accurate effects of evolution. However, by using such a set-up, we would have potentially failed to identify masked phenotypic responses as the ones observed in our study. Identifying such masked responses is important in the light of evaluating species’ responses to environmental change, especially when species evolve within complex communities^17,18^.

Abrupt exposure to a novel abiotic environmental condition—like salinity in our study—may exert strong directional selection reducing population’s fitness, resulting in a strong shift in the trait mean^54^, and even cause extinction (as was the case for *T. thermophila*). Klironomos et al.^55^ demonstrated that abrupt environmental exposure often results in an overestimation of community responses to environmental change. Our results support a similar conclusion regarding the importance of the magnitude of the abiotic environmental change. For two traits—biomass and dispersal ability—*P. aurelia* showed a distinct genetic trait change between the zero and highest salinity. This indicates that not only differences between a single-step versus gradual exposure might change species responses, but also the magnitude of the abiotic environmental change. However, *P. aurelia*’s traits did not differ between the highest salinity in the absence of competition. It did, however, when competing species were present. The effect size of genetic trait change in response to salinity of *P. aurelia* was largest in the highest salinity of the competition treatment. This result shows that competition altered the genetic response to salinity, with greatest effect being observed when the focal species was exposed to the largest abiotic change. Future studies should evaluate whether the community setting would have similar effects in response to gradually increasing salinity.

Our results show that understanding species’ genetic and plasticity responses to both abiotic and biotic environments will improve our ability to make successful predictions on species persistence, helping foresee the winners and losers of global change. Communities consist of different species which all simultaneously respond to ongoing changes in the environment. From our study and others, it is clear that species differ in plastic and genetic responses^39,56^, ultimately determining species persistence. One of our species—*P. Aurelia*—showed clear genetic and plastic responses to the abiotic change, with alterations in these responses when this species evolved in the presence of competing species. The other species—*S. teres*—however showed only limited responses with no clear pattern in its genetic and plastic responses and did not persist. Even though in species-rich communities the biotic context will be far more complex than what we addressed experimentally—with species abundances and the strength of species interactions fluctuating over time—we believe that our results provide a step toward understanding species responses in complex communities. In order to persist, species must be capable of adapting not only to the abiotic conditions but also to their biotic environment.

## Methods

To test for species’ plastic and genetic responses to environmental change, and how these responses mediate species persistence when embedded in a community of competing species, we performed controlled microcosm experiments using three competing freshwater ciliate species along an increasing salinity gradient. Specifically, the three species were evolved in the absence (only one species present) or presence (all three species together) of competition for a selection period of 3 months. After the evolution experiment, a common garden was performed to assess plastic and evolutionary responses to salinity and how such responses were altered by competition (Fig. 1).

### Model organism

We used aquatic ciliates as a model system. Aquatic ciliates are globally distributed and found in almost all freshwater ecosystems^57^. They are well-established model organisms to test ecological and evolutionary theories^52,58–60^, as they are easy to quantify, manipulate, and have short generation times^29^. Specifically, we used *Paramecium aurelia*^61^, *Spirostomum teres*^62^, and *Tetrahymena thermophila*^63^. These species naturally co-occur, for example in temperate ponds^28^. All three species reproduced asexually throughout the experiment, and have reported generation times of about 11 h for *T. thermophila*, 28 h for *P. aurelia*, and 42 h for *S. teres*^52^. All species were kept in autoclaved protozoan pellet medium, made of local spring water and 0.46 g/l of Protozoan Pellets (Carolina Biological Supply, NC USA) and 5% bacterial inoculum which consists of three bacterial species (*Bacillus subtilis*, *Brevibacillus brevis,* and *Serratia fonticola*; obtained from Carolina Biological Supply)^29^. The Protozoan Pellets provide nutrients for the added bacteria. The bacterial inoculum was prepared 2 days before the set-up of the experiment, in which we added a volume of 1 ml of equal densities of the three bacterial species to autoclaved protozoan pellet medium and let them grow for 48 h.

### Experimental evolution

We used a full-factorial design of species kept in the absence and presence of competing species along five different salinity environments (adding appropriate levels of NaCl to the protist medium to reach concentrations of 0, 0.5, 1, 2, and 4 g/l respectively). These NaCl concentrations correspond to a natural, unpolluted freshwater pond or lake (0 g/l), as well as realistic and currently experienced as well as future predicted environmentally relevant NaCl concentrations^64,65^. The experiment was conducted in 50 ml centrifuge tubes. The tubes were filled to a total volume of 25 ml. Bacteria inoculum (5% of the volume) and protists cultures (65% of the volume) were added at carrying capacity. For the competition treatment, species were added at equal densities.

Each replicate contained populations of *P. aurelia*, *S. teres* and *T. thermophila* either alone (i.e. absence of interspecific competition) or all three together (i.e. presence of interspecific competition) across the five different salt concentrations. The species came from lab stock cultures maintained such they contained standing phenotypic and genotypic variation. Each combination of a particular salt concentration with either absence or presence of competing species was replicated three times, resulting in a total of 60 microcosms. These were led to evolve for 78 days under constant temperature of 20 °C and constant light intensity of 18.21 µmol/m^2^/s. During the length of the experimental evolution, 5% of the volume of the replicate was replaced daily with fresh protozoan medium with the corresponding salinity. At the end of the experimental evolution, on day 78, a common garden experiment was initiated (detailed next section). The populations obtained at the end of the selection phase are further referred to as the selected populations. We omitted *T. thermophila* from the common garden and subsequent analysis, as it went extinct in almost all microcosms during the evolution experiment.

### Common garden experiment

The goal of the common garden experiment was to (1) test whether species evolved in competition showed a different response to the abiotic environment (salinity) and (2) separate evolutionary from plastic effects to salinity and competing species. In a common garden experiment, populations of the same species evolved in different selection environments are placed in a common environment^66^. We performed a common garden experiment for each selected population using multiple salinity common garden environments. Since we use multiple common environments, we could quantify whether the response to salinity depends on interspecific competition and the salt concentration the population was selected in (Fig. 1). Because species obtained from the competition treatment could not be separated upon inoculation of the common garden, plastic and genetic effects to salinity could be separated only for those replicates in the absence of competition and for those in which *S. teres* went extinct.

The common garden microcosms were hosted in 50 ml centrifuge tubes. They were inoculated at day 78 as in the evolution experiment, and traits were measured on day 82. They were filled with 24 ml of protist medium with the adequate salt concentration, such that when common gardens were inoculated with 1 ml from the microcosms obtained of the experimental evolution it resulted in a focal salt concentration of 0, 0.5, 1, 2, and 4 NaCl g/l, respectively. For each microcosm obtained from the experimental evolution used for the common garden, two replicated microcosms were filled for the common garden phase. Because of the impossibility of handling aquatic ciliates without handling small amounts of medium, one cannot strictly inoculate the non-zero salinity selection lines into the zero-salinity common garden. Thus, for the non-zero salinity selection treatments, we only had 4 respective common gardens (Fig. 1). In total, this resulted in 378 microcosms.

### Data collection and pre-analysis

Microcosms were sampled 4 days after the start of the selection experiment, and on the day of the common garden before inoculation of the common garden (day 78), each time by taking a sample of 500 µl. The common garden experiment was sampled 4 days after inoculation, extracting two samples of 250 µl each on day 82. Video recording and automated analysis were used to collect data on population densities (number of cells) and traits (bio-area, aspect ratio, and gross speed)^67,68^. We recorded 20 s videos (25 frames per second) of an effective sampled volume of 34.4 µl using a Leica M205C stereomicroscope at a 16-fold magnification and an Orca Flash 4 camera (Hamamatsu). Videos were analyzed using a customized version of the Bemovi package in R (available on https://github.com/efronhofer/bemovi^67^).

Data output of the videos was visually inspected, and upon analysis, data entries reflecting *P. aurelia* or *S. teres* particles with a bio-area smaller than 1000 µm^2^ were removed from the data as these reflected non-living particles that were accidentally tracked. After species identification, videos were again visually inspected to ensure the identification was performed correctly. Trait values of those videos that contained only one of the species were manually assigned to the correct species identity when needed.

### Statistical analysis

In this study, we focused on three key traits: biomass [µm^2^] (i.e. area calculated from the major and minor axis of an individual), cell shape (i.e. ratio of the major to minor cell axis of an individual) and dispersal ability [µm/s] (i.e. the gross speed of an individual). First, we evaluated the temporal shift observed in these traits across the different salt concentrations, and whether competition altered this shift. Second, we performed a common garden experiment to (1) evaluate whether the response to salinity depends on interspecific competition and salt concentration the species was evolved in, and (2) quantify plastic and genetic components to trait changes.

All statistical analyses were performed using linear mixed-effect models implemented in the R package ‘lmerTest’^69^. All models were inspected for constant variance and linear fit by fitting residuals against the model estimates, for normality of the model residuals using a q–q plot, and for outliers using cook's distance plots. R^2^ values were calculated as described in Nakagawa et al.^70^ using the function ‘r.squaredGLMM’ implemented in the package ‘MuMIn’^71^. We calculated a marginal R^2^ value (${R}_{m}^{2}$) representing the variance explained by the fixed effects and a conditional R^2^ value (${R}_{c}^{2}$) reflecting the variance explained by the total model. Specifically, these were calculated as follows:

$${R}_{m}^{2}=\frac{{\sigma }_{f}^{2}}{{\sigma }_{f}^{2}+ {\sigma }_{r}^{2}+ {\sigma }_{e}^{2}}$$

$${R}_{c}^{2}=\frac{{\sigma }_{f}^{2}+{\sigma }_{r}^{2}}{{\sigma }_{f}^{2}+ {\sigma }_{r}^{2}+ {\sigma }_{e}^{2}}$$

with ${\sigma }_{f}^{2}$ the variance of the fixed effects, ${\sigma }_{r}^{2}$ the variance of the random effects and ${\sigma }_{e}^{2}$ the observation-level variance. All analyses were conducted in R version 3.5.2.

#### Temporal trait shift along environmental (salinity) gradient (experimental evolution)

To quantify the trait shift observed during the selection phase and how this was altered by competition (comparison of Fig. 1a,b in the main text), we calculated the trait difference of all pairwise combinations within replicates between individuals at the start (day 4) and end (day 78) of the selection phase. In addition, we performed a linear mixed effect model on the total data combining all salinity by competition treatments. The structure of the linear mixed effect model mirrors the structure of the experiment. We used the trait of interest (bio-area, aspect ratio, or gross speed) as a response variable (*Y*), while the fixed effects were the salt concentration (‘salinity’), the measurement date (indicated by ‘time’, a binary variable reflecting start and end of the evolution experiment), whether the species was evolved in the absence or presence of competing species (indicated by the variable ‘competition’), density and bio-fraction of the other species. Density was included, because previous studies have shown the possible importance of intraspecific density effects^72^. Bio-fraction reflects the relative biomass of the competing species, and thus reflects the different species compositions among treatments and replicates. The microcosm ID was included as a random factor in the model. We also included the two and three-way interactions between salinity, time and competition. We can formalize the model as:

$${\text{Y }}\sim \alpha_{{\text{i}}} + \beta_{{\text{s}}} \cdot {\text{salinity}} + \beta_{{\text{t}}} \cdot {\text{time }} + \beta_{{\text{c}}} \cdot {\text{competition }} + \beta_{{\text{d}}} \cdot {\text{density }} + \beta_{{{\text{bf}}}} \cdot {\text{biofraction }} + \gamma_{{{\text{s}} \times {\text{t}}}} \cdot {\text{salinity}} \times {\text{time }} + \gamma_{{{\text{s}} \times {\text{c}}}} \cdot {\text{salinity}} \times {\text{competition }} + \gamma_{{{\text{t}} \times {\text{c}}}} \cdot {\text{time}} \times {\text{competition }} + \gamma_{{{\text{s}} \times {\text{t}} \times {\text{c}}}} \cdot {\text{salinity}} \times {\text{time}} \times {\text{competition}} + \varepsilon ,$$

with α_i_ the random intercept for microcosm ID *i*, β_X_ the regression coefficient for variable *X*, γ_X×Z_ the regression coefficient of the two-way interaction between variables *X* and *Z*, and γ_X×Z×T_ the three-way interaction between variables *X*, *Z* and *T*. A significant effect of time reflects a trait change from start to end of the evolution experiment. A significant interaction between time and competition reflects that the trait change differs between the absence and presence of competing species.

## Phenotypic response to salinity and competition (common garden)

To quantify whether interspecific competition altered a species’ response to salinity—both in which it was evolved in (referred to as ‘historical salinity’) and in which it was exposed to during the common garden experiment (referred to as ‘common garden salinity environment’), we fitted a linear mixed-effect model. In this model, the phenotypic trait of interest was included as the response variable, while historical salinity and common garden salinity environment, whether the species was evolved in the presence and absence of competing species (indicated by the variable ‘competition’), density, and the bio-fraction of the other species were included as fixed effects. The variable bio-fraction reflects the relative biomass of the competing species. It was incorporated to correct for the presence of the other species. Microcosm ID from the selection phase and replicate were included as random factors, the latter nested within the interaction of historical salinity and common garden salinity environment. Two-way and three-way interactions between historical salinity, common garden (CG) salinity environment and competition were also included. Specifically, we formalize the model as:

$${\text{Y}}\sim \alpha_{{{\text{ij}}}} + \beta_{{{\text{so}}}} \cdot {\text{historical salinity }} + \beta_{{{\text{sd}}}} \cdot {\text{CG salinity }} + \beta_{{\text{c}}} \cdot {\text{competition }} + \beta_{{\text{d}}} \cdot {\text{density }} + \beta_{{{\text{bf}}}} \cdot {\text{biofraction }} + \gamma_{{{\text{so}} \times {\text{sd}}}} \cdot {\text{historical salinity}} \times {\text{CG salinity }} + \gamma_{{{\text{so}} \times {\text{c}}}} \cdot {\text{historical salinity}} \times {\text{competition }} + \gamma_{{{\text{sd}} \times {\text{c}}}} \cdot {\text{CG salinity}} \times {\text{competition }} + \gamma_{{{\text{so}} \times {\text{sd}} \times {\text{c}}}} \cdot {\text{historical salinity}} \times {\text{CG salinity}} \times {\text{competition }} + \varepsilon ,$$

with α_ij_ being the random intercept for microcosm ID *i* and replicate *j*, β_X_ the regression coefficient for variable *X*, γ_X×Z_ the regression coefficient of the two-way interaction between variables *X* and *Z*, and γ_X×Z×T_ the three-way interaction between variables *X*, *Z* and *T*. A significant interaction between competition and CG salinity reflects a difference in the direct response to salinity when species evolved in the absence or presence of competing species. A significant interaction between historical competition and historical salinity reflects whether the historical response to salinity—salinity conditions in which the species was evolved in—differs between the competition treatments. Note that common garden microcosms from the competition treatment also contain the competitor species. Hence, trait values obtained from those microcosms not only reflect a response to salinity but also to the presence of the competing species.

### Robustness analysis

To test the robustness of our statistical results, we performed a sensitivity analysis. Specifically, we bootstrapped the data without replacement on increasingly smaller fractions of our data (from 90 to 10%). For each bootstrap sample, a linear mixed-effect model was conducted exactly as described in the previous section. For each sample, model regression coefficients and their corresponding p value were stored. After 1000 bootstrap samples, mean effect sizes and mean p values with 95% confidence intervals were calculated. Supplementary Figs. S3 and S4 show the robustness analysis on the linear mixed effect model of phenotypic response to salinity and competition for *P. aurelia* and *S. teres*.

## Genetic and plastic response to salinity (common garden)

We used a previously developed method—the reaction norm approach^15,46^—to quantify contributions of (ancestral) plasticity, mean trait evolution and evolution of plasticity to observed trait changes from our ancestral population of the selection phase (at day 4) to each of the selected populations (i.e. the populations evolved in the different abiotic × biotic conditions) measured in the common garden. Specifically, we used trait values collected from the ancestral population in the absence of competition and partitioned observed trait changes from this population to each of the selected populations evolved in the absence and presence of competing species. Using trait values of the ancestral population of the competition treatment would confound plasticity responses to the presence of competing species.

The components of the reaction norm approach relate to linear regression coefficients of a linear regression model with interaction term when using indicator variables^73,74^. Therefore, instead of using the mathematical formulae for these components, we estimated contributions of (ancestral) plasticity, mean trait evolution and evolution of plasticity using a linear regression model with two indicator variables; one describing whether trait values are obtained from the ancestral or from one of the selected populations, and one describing whether trait values are obtained from the 0.5 g/l salt environment used in the common garden experiment or either from the 1, 2 or 4 g/l salt environment used in the common garden experiment. In words, the first indicator variable describes trait change between the ancestral and one of the selected populations in the 0.5 g/l salt environment, and thus reflects genetic trait change. In the regression model this variable had two factorial states: one referring to the ancestral population, and one referring to the selected population. The second indicator variable describes trait change of the ancestral population between different salinity conditions used in the common garden experiment, and thus reflects a plasticity response to salinity of this ancestral population. In the regression model this variable had two factorial states: one referring to the 0.5 g/l salt environment and one referring to either 1, 2 and 4 g/l salt environment. The interaction term then describes the change in the plasticity response to salinity between the ancestral and the descendant population, and thus reflects an evolution of plasticity component.

Plasticity, mean trait evolution and evolution of plasticity were estimated using a linear mixed effect model with each trait of interest as response variable, and with the two previously described indicator variables as fixed effects. In addition, we also included density as a fixed covariate to correct for density differences between the samples and included unique microcosm ID as random effect. This model was calculated separately for each selected population, and for the 1 and 2 g/l salt environment of the common garden. Plastic and genetic components could not be calculated for the 4 g/l salt environment, as we had no trait values of the ancestral population in the absence of competing species in that salt environment. Results from the reaction norm analysis can be found in Supplementary Fig. S5 for *P. aurelia* and Supplementary Fig. S6 for *S. teres*. Reaction norms visualizing these changes are given in Supplementary Fig. S7 for *P. aurelia*, and in Supplementary Fig. S8 for *S. teres*. Results of the statistical analysis are given in Supplementary Tables S5-S8.

Important to note is that comparisons with selected populations from the competition treatment may not reflect pure genetic changes, as trait values of those populations were obtained in the presence of other species and may thus be confounded with a plasticity response to competition. However, for the higher salt concentrations (2 and 4 g/l), *S. teres* went extinct in all replicates except one of the competition treatment in the 4 g/l salinity. Thus, for these two conditions, the component ‘mean trait evolution’ does accurately reflect genetic change. Calculations were repeated with omitting replicate ID 120, as during screening a single *S. teres* individual was found (Supplementary Fig. 9).

Lastly, as the reaction norm analysis estimates evolution of plasticity, we additionally ran linear mixed effect models to estimate the phenotypic plasticity response of each selected population. Specifically, we included the trait of interest as response variable and the salinity conditions for which a linear plasticity response was estimated as fixed effect. For example, if we estimated the plasticity response to the 1 g/l salinity of the selected population evolved in the zero salinity environment, we included trait values of that selected population of the 0.5 and 1 g/l common garden environments. Additionally, in the mixed effect model we included density as a fixed covariate, and the unique microcosm ID as a random factor. Results of this statistical analysis are given in Figs. 4 and 5, and Supplementary Tables S9–S12.

## Genetic differences of *P. aurelia* between the absence and presence of competing species (common garden)

To evaluate whether interspecific competition could alter the evolutionary response of species, we compared populations evolved in the presence and absence of competition, but selected in the same salinity condition. However, this requires that trait values of the evolved populations in the absence and presence of competition are measured without competitor species. We were able to quantify these responses for the *P. aurelia* populations from the two highest salinity conditions for which *S. teres* went extinct. The extinction of *S. teres* allowed to attribute the trait differences found in the two highest salinity conditions to genetic trait differences. We ran a linear mixed effect model comparing *P. aurelia* selected populations evolved in the same salinity condition but differing in the competition treatment for each common garden salinity environment. Specifically, in the linear mixed effect model each trait of interest was used as response variable, and competition as predictor. We included density as a covariate, and replicate and microcosm ID as random factors. Results of this statistical analysis are given in Fig. 5 main text, and Supplementary Table S13. Similarly, we ran a linear mixed effect model to compare trait values of *P. aurelia* populations evolved in the lower salinity conditions in the absence and presence of competition. Results of this statistical analysis are given in Supplementary Fig. S9 and Supplementary Table S14.

## Supplementary Information

Supplementary Information.

## Data Availability

The data is available at the Dryad Digital Repository  with DOI number: 10.5061/dryad.t4b8gtj28.

## Code availability

The code is available at the Dryad Digital Repository  with DOI number: 10.5061/dryad.t4b8gtj28.
