# Supplementary material for: Competition alters species’ plastic and genetic response to environmental change
Source: Sci Rep. 2021 Dec 7;11:23518. doi: 10.1038/s41598-021-02841-8 (PMC8651732; doi:10.1038/s41598-021-02841-8)
Supplement: Supplementary file 1 — Supplementary Information. [file 41598_2021_2841_MOESM1_ESM.pdf]

Supporting Information to "Competition alters species' plastic and genetic response to environmental change"

Lynn Govaert[1,2,3,*], Luis J. Gilarranz[2], Florian Altermatt[1,2,3]

[1]Department of Evolutionary Biology and Environmental Studies, University of Zurich, Winterthurerstrasse 190, 8057 Zürich, Switzerland

[2]Department of Aquatic Ecology, Eawag: Swiss Federal Institute of Aquatic Science and Technology, Überlandstrasse 133, 8600 Dübendorf, Switzerland

[3]URPP Global Change and Biodiversity, University of Zurich, Winterthurerstrasse 190, 8057 Zurich

*Corresponding author: lynn.govaert@igb-berlin.de. Current address: Leibniz-Institute of Freshwater Ecology and Inland Fisheries (IGB), Müggelseedamm 310, 12587 Berlin, Germany

This file contains supplementary tables:

- Table S1: Summary output regression analysis of the phenotypic response during the selection phase for *Paramecium aurelia* reared in the absence and presence of competing species.

- Table S2: Summary output regression analysis of the phenotypic response during the selection phase for *Spirostomum teres* reared in the absence and presence of competing species.

- Table S3: Summary regression analysis for the combined data set of *Paramecium aurelia* reared in the absence and presence of competing species during the common garden.

- Table S4: Summary regression analysis for the combined data set of *Spirostomum teres* reared in the absence and presence of competing species from the common garden.

- Table S5: Summary regression analysis for the partitioning of (ancestral) plasticity, mean trait evolution and evolution of plasticity of temporal trait change in *Paramecium aurelia* between the ancestral population of the selection phase (day 4) and each selected population evolved in the absence of competing species (given by historical salinity, i.e. the salinity used during the selection phase).

- Table S6: Summary regression analysis for the partitioning of (ancestral) plasticity, mean trait evolution and evolution of plasticity of temporal trait change in *Paramecium aurelia* between the ancestral population of the selection phase (day 4) and each selected population evolved in the presence of competing species (given by historical salinity, i.e. the salinity used during the selection phase).

- Table S7: Summary regression analysis for the partitioning of (ancestral) plasticity, mean trait evolution and evolution of plasticity of temporal trait change in *Spirostomum teres* between the ancestral population of the selection phase (day 4) and each selected population evolved in the absence of competing species (given by historical salinity, i.e. the salinity used during the selection phase).

- Table S8: Summary regression analysis for the partitioning of (ancestral) plasticity, mean trait evolution and evolution of plasticity of temporal trait change in *Spirostomum teres* between the ancestral population of the selection phase (day 4) and each selected population evolved in the presence of competing species (given by historical salinity, i.e. the salinity used during the selection phase).

- Table S9: Summary of regression analysis for the phenotypic plasticity response to salinity of *Paramecium aurelia* evolved in the absence of competing species.

- Table S10: Summary of regression analysis for the phenotypic plasticity response to salinity of *Paramecium aurelia* evolved in the presence of competing species.

- Table S11: Summary of regression analysis for the phenotypic plasticity response to salinity of *Spirostomum teres* evolved in the absence of competing species.

- Table S12: Summary of regression analysis for the phenotypic plasticity response to salinity of *Spirostomum teres* evolved in the presence of competing species.

- Table S13: Genetic trait difference for high salinity selected *Paramecium aurelia* populations comparing those evolved in the absence and presence of competing species.

- Table S14: Trait difference due to historical competition and the presence of competitors for the salinity selected *Paramecium aurelia* populations evolved in the 0, 0.5 and 1 g/l salt conditions.

and supplementary figures:

- Figure S1: Temporal phenotypic difference during the selection phase (a-c) *Paramecium aurelia* and (d-f) *Spirostomum teres* along salinity and between populations in the absence and presence of competing species.

- Figure S2: Common garden results of *Paramecium aurelia* and *Spirostomum teres* visualized as reaction norms in the absence and presence of competition.

- Figure S3: Robustness analysis for the effect sizes and their corresponding p-values obtained from regression analysis on the common garden data for *Paramecium aurelia.*

- Figure S4: Robustness analysis for the effect sizes and their corresponding p-values obtained from regression analysis on the common garden data for *Spirostomum teres.*

- Figure S5: Reaction norm analysis for *Paramecium aurelia* quantifying trait change in biomass, cell shape and dispersal between the ancestral and each selected population.

- Figure S6: Reaction norm analysis for *Spirostomum teres* quantifying trait change in biomass, cell shape and dispersal between the ancestral and each selected population.

- Figure S7: Phenotypic plasticity response to salinity of *Paramecium aurelia* for biomass, cell shape and dispersal ability of the ancestral (Anc.) and each of the selected (0, 0.5, 1, 2 and 4 g/l) populations evolved in the absence and presence of competitors.

- Figure S8: Phenotypic plasticity response to salinity of *Spirostomum teres* for biomass, cell shape and dispersal ability of the ancestral (Anc.) and each of the selected (0, 0.5, 1, 2 and 4 g/l) populations evolved in the absence and presence of competitors.

- Figure S9: Trait difference due to historical competition and the presence of competitors for the salinity selected *Paramecium aurelia* populations evolved in the 0, 0.5 and 1 g/l salt conditions.

- Figure S10: Biomass and cell shape values for *Paremecium aurelia* and *Spirostomum teres* obtained in the common garden of those populations evolved in the absence of competition along the salinity conditions used in the selection phase.

- Figure S11: Community composition at the start (day 4) and end (day 78) of the experimental evolution and the common garden (day 82).

- Figure S12: Genetic trait difference for the highest salinity selected *Paramecium aurelia* populations comparing those evolved in the absence and presence of competing species including microcosm ID 120.

Supplementary Tables

Table S1: Summary output regression analysis of the phenotypic response during the selection phase for *Paramecium aurelia* reared in the absence and presence of competing species. Regression slope (β) ± standard error (SE), degrees of freedom (df), test statistic (t-value) and p-value are given for traits biomass (quantified as bio-area), cell shape (quantified as aspect ratio), and dispersal ability (quantified as gross speed) for *Paramecium aurelia* reared in the absence and presence of competing species during the selection phase. A total of n = 3084 data points were used for this analysis. Significant effects ($p < 0.05$) are highlighted in bold. R^2 values report the variation explained by the fixed effects (R^2_{fixed}) and by both fixed and random effects (R^2_{total}) for the three traits (detailed in Methods).

Biomass	$\beta \pm$ SE	df	t-value	p-value
Salinity	-9.296 ± 82.220	39.485	-0.113	0.911
Competition	162.860 ± 137.175	28.062	1.187	0.245
**Time**	-2039.762 ± 125.427	367.150	-16.262	< **0.001**
Biofraction Spite	-252.370 ± 259.228	2772.703	-0.974	0.330
**Density**	-4.561 ± 2.204	123.469	-2.069	**0.041**
Salinity × Competition	73.807 ± 95.016	38.791	0.777	0.442
Salinity × Time	71.913 ± 87.121	120.186	0.825	0.411
**Competition × Time**	-509.507 ± 139.335	2210.023	-3.657	< **0.001**
Sal × Competition × Time	-36.765 ± 104.423	237.562	-0.352	0.725
R^2_{fixed}=0.4680, R^2_{total}=0.4931				
Cell shape	$\beta \pm$ SE	df	t-value	p-value
Salinity	5.383e-02 ± 4.192e-02	4.949e+01	1.284	0.205
Competition	7.119e-02 ± 7.779e-02	2.352e+01	0.915	0.369
**Time**	4.131e-01 ± 5.041e-02	6.799e+02	8.195	< **0.001**
Biofraction Spite	-9.559e-02 ± 9.566e-02	3.041e+03	-0.999	0.318
Density	1.090e-03 ± 9.366e-04	3.888e+02	1.164	0.245
Salinity × Competition	-7.845e-02 ± 4.944e-02	3.957e+01	-1.587	0.121
**Salinity × Time**	-9.294e-02 ± 3.820e-02	1.962e+02	-2.433	**0.016**
**Competition × Time**	-3.608e-01 ± 5.223e-02	2.370e+03	-6.907	< **0.001**
**Sal × Competition × Time**	1.309e-01 ± 4.361e-02	3.241e+02	3.003	**0.003**
R^2_{fixed}=0.0592, R^2_{total}=0.1798				
Dispersal ability	$\beta \pm$ SE	df	t-value	p-value
Salinity	-8.081 ± 22.831	54.727	-0.354	0.725
Competition	35.140 ± 40.886	28.603	0.859	0.397
**Time**	-150.905 ± 29.232	624.694	-5.162	< **0.001**
Biofraction Spite	94.333 ± 56.589	3014.059	1.667	0.100
**Density**	-2.514 ± 0.538	317.907	-4.673	< **0.001**
Salinity × Competition	-44.292 ± 26.691	46.310	-1.659	0.104
Salinity × Time	1.114 ± 21.679	186.356	0.051	0.960
**Competition × Time**	-144.973 ± 30.756	2379.796	-4.714	< **0.001**
**Sal × Competition × Time**	72.304 ± 25.008	319.101	2.891	**0.004**
R^2_{fixed}=0.0781, R^2_{total}=0.1700				

Table S2: Summary output regression analysis of the phenotypic response during the selection phase for *Spirostomum teres* reared in the absence and presence of competing species. Regression slope (β) ± standard error (SE), degrees of freedom (df), test statistic (t-value) and p-value are given for traits biomass (quantified as bio-area), cell shape (quantified as aspect ratio), and dispersal ability (quantified as gross speed) for *Spirostomum teres* reared in the absence and presence of competing species during the selection phase. A total of n = 1294 data points were used for this analysis. Significant effects ($p < 0.05$) are highlighted in bold. R^2 values report the variation explained by the fixed effects (R^2_{fixed}) and by both fixed and random effects (R^2_{total}) for the three traits (detailed in Methods).

Biomass	β	df	t-value	p-value
**Salinity**	610.58 ± 223.57	53.72	2.731	**0.009**
Competition	-261.84 ± 556.69	55.13	-0.470	0.640
**Time**	1592.29 ± 252.09	1184.60	6.316	< **0.001**
Biofraction Pau	1408.29 ± 1846.79	463.97	0.763	0.446
Density	-11.76 ± 6.17	192.47	-1.906	0.058
Salinity × Competition	-662.76 ± 430.55	63.13	-1.539	0.129
**Salinity × Time**	-1055.92 ± 184.46	587.67	-5.724	< **0.001**
**Competition × Time**	-1967.91 ± 901.09	982.65	-2.184	**0.029**
**Sal × Competition × Time**	2236.24 ± 912.71	621.81	2.450	**0.015**
R^2_{fixed}=0.049, R^2_{total}=0.133				

Cell shape	β ± SE	df	t-value	p-value
Salinity	-1.814e-01 ± 1.845e-01	6.215e+01	-0.984	0.329
Competition	-5.602e-01 ± 4.593e-01	6.164e+01	-1.220	0.227
**Time**	1.670e+00 ± 2.054e-01	1.207e+03	8.130	< **0.001**
Biofraction Pau	-2.883e+00 ± 1.509e+00	5.167e+02	-1.911	0.057
**Density**	-1.162e-02 ± 5.056e-03	2.265e+02	-2.297	**0.023**
Salinity × Competition	3.099e-01 ± 3.549e-01	7.025e+01	0.873	0.385
Salinity × Time	-1.061e-01 ± 1.506e-01	6.453e+02	-0.705	0.481
**Competition × Time**	1.686e+00 ± 7.346e-01	1.026e+03	2.295	**0.022**
**Sal × Competition × Time**	-1.970e+00 ± 7.451e-01	6.735e+02	-2.644	**0.008**
R^2_{fixed}=0.1507, R^2_{total}=0.2297				

Dispersal ability	β ± SE	df	t-value	p-value
Salinity	7.6202 ± 12.9847	40.5482	0.587	0.561
Competition	51.8668 ± 32.5373	52.8887	1.594	0.117
**Time**	285.5693 ± 15.8943	1048.4906	17.967	< **0.001**
Biofraction Pau	-130.0620 ± 114.2791	362.6806	-1.138	0.256
**Density**	-2.5573 ± 0.3736	120.2324	-6.845	< **0.001**
Salinity × Competition	-14.6756 ± 25.3208	62.3073	-0.580	0.564
**Salinity × Time**	-56.2762 ± 11.4629	455.8687	-4.909	< **0.001**
Competition × Time	-87.0554 ± 56.5613	849.8959	-1.539	0.124
Sal × Competition × Time	-46.0942 ± 56.8212	534.9767	-0.811	0.418
R^2_{fixed}=0.3061, R^2_{total}=0.3482				

Table S3-a: Summary regression analysis for the combined data set of *Paramecium aurelia* reared in the absence and presence of competing species during the common garden. Regression slope (β) $\pm$ standard error (SE), degrees of freedom (df), test statistic (t-value) and p-value are given for biomass (quantified as bio-area), cell shape (quantified as cell size ratio), and dispersal ability (quantified as gross speed) for *P. aurelia* reared in the absence and presence of competition obtained during the common garden. Historical salinity (Hist. sal.) refers to the salinity used in the selection phase. Historical competition (Comp.) refers to the absence or presence of competing species in the selection phase. Common garden salinity environment (CG sal.) refers to the salinity used in the common garden. Density refers to the intraspecific density of the species. Biofraction (Biofrac.) *S. teres* refers to the proportional biomass of the competitor species *S. teres.* A total of $n = 7533$ data points were used for this analysis. Significant effects ($p < 0.05$) are highlighted in bold. R^2 values report the variation explained by the fixed effects (R^2_{fixed}) and by both fixed and random effects (R^2_{total}) (detailed in Methods).

Biomass	$\beta \pm$ SE	df	t-value	p-value
Hist. sal.	80.857 ± 65.562	38.054	1.233	0.225
**CG sal.**	165.631 ± 53.105	46.339	3.119	**0.003**
**Comp.**	372.819 ± 174.624	26.984	2.135	**0.042**
Biofrac. *S. teres*	-22.890 ± 167.878	5872.744	-0.136	0.892
**Density**	-5.023 ± 2.114	3742.823	-2.376	**0.018**
Hist. sal. × CG sal.	-11.384 ± 18.681	37.880	-0.609	0.546
**Hist. sal. × Comp.**	-193.950 ± 83.473	25.474	-2.324	**0.028**
CG sal. × Comp.	-32.778 ± 37.960	7119.154	-0.863	0.388
Hist. sal. × CG sal. × Comp.	5.151 ± 13.418	7510.317	0.384	0.701
R^2_{fixed}=0.0455, R^2_{total}=0.2010				

Cell shape	$\beta \pm$ SE	df	t-value	p-value
Hist. sal.	0.002 ± 0.031	52.300	0.055	0.956
**CG sal.**	-0.182 ± 0.037	37.758	-4.936	< **0.001**
**Comp.**	-0.481 ± 0.067	34.115	-7.207	< **0.001**
Biofrac. *S. teres*	0.045 ± 0.101	4482.007	0.440	0.660
**Density**	-0.004 ± 0.001	2737.770	-3.294	**0.001**
Hist. sal. × CG sal.	0.008 ± 0.013	31.185	0.581	0.566
**Hist. sal. × Comp.**	0.122 ± 0.031	29.614	3.923	< **0.001**
**CG sal. × Comp.**	0.109 ± 0.023	7315.650	4.715	< **0.001**
Hist. sal. × CG sal. × Comp.	-0.015 ± 0.008	7508.625	-1.873	0.061
R^2_{fixed}=0.1335, R^2_{total}=0.2110				

Table S3-b: CONTINUE TABLE S3

Dispersal ability	$\beta \pm$ SE	df	*t*-value	*p*-value
Hist. sal.	3.762 ± 28.739	56.436	0.131	0.896
**CG sal.**	-195.564 ± 34.060	49.993	-5.742	< **0.001**
**Comp.**	-228.782 ± 64.216	40.982	-3.563	**0.001**
Biofrac. *S. teres*	-87.498 ± 96.953	4578.145	-0.902	0.367
**Density**	-12.349 ± 1.216	2804.397	-10.151	< **0.001**
Hist. sal. × CG sal.	20.313 ± 12.126	41.186	1.675	0.101
**Hist. sal. × Comp.**	79.097 ± 30.022	35.674	2.635	**0.012**
**CG sal. × Comp.**	141.002 ± 22.163	7306.961	6.362	< **0.001**
**Hist. sal. × CG sal. × Comp.**	-37.060 ± 7.827	7510.953	-4.735	< **0.001**
R^2_{fixed}=0.1112, R^2_{total}=0.1880				

Table S4-a: Summary regression analysis for the combined data set of *Spirostomum teres* reared in the absence and presence of competing species during the common garden. Regression slope (β) ± standard error (SE), degrees of freedom (df), test statistic (t-value) and p-value are given for biomass (quantified as bio-area), cell shape (quantified as cell size ratio), and dispersal ability (quantified as gross speed) for *S. teres* reared in the absence and presence of competition obtained during the common garden. Historical salinity (Hist. sal.) refers to the salinity used in the selection phase. Historical competition (Comp.) refers to the absence or presence of competing species during the selection phase. Common garden salinity environment (CG sal.) refers to the salinity used in the common garden. Density refers to the intraspecific density of the species. Biofraction (Biofrac.) *P. aurelia* refers to the proportional biomass of the competitor species *P. aurelia*. A total of $n = 1390$ data points were used for this analysis. Significant effects ($p < 0.05$) are highlighted in bold. R^2 values report the variation explained by the fixed effects (R^2_{fixed}) and by both fixed and random effects (R^2_{total}) for the three traits (detailed in Methods).

Biomass	β	df	t-value	p-value
Hist. sal.	-347.106 ± 809.848	17.288	-0.429	0.674
CG sal.	-14.446 ± 182.467	46.711	-0.079	0.937
Comp.	365.062 ± 1393.689	113.017	0.262	0.794
**Biofrac. *P. aurelia***	-2882.272 ± 1093.457	531.744	-2.636	**0.009**
Density	-45.023 ± 27.491	355.691	-1.638	0.102
Hist. sal. × CG sal.	320.486 ± 240.219	35.150	1.334	0.191
Hist. sal. × Comp.	-1701.200 ± 1724.591	67.551	-0.986	0.327
CG sal. × Comp.	598.296 ± 575.862	1203.044	1.039	0.299
Hist. sal. × CG sal. × Comp.	-392.069 ± 986.731	1048.913	-0.397	0.691
R^2_{fixed}=0.0956, R^2_{total}=0.2263				

Cell shape	β ± SE	df	t-value	p-value
Hist. sal.	0.691 ± 0.605	17.293	1.141	0.269
CG sal.	-0.079 ± 0.114	48.669	-0.697	0.489
Comp.	1.216 ± 0.852	71.022	1.427	0.158
Biofrac. *P. aurelia*	-0.723 ± 0.615	761.608	-1.176	0.240
Density	0.017 ± 0.015	723.380	1.117	0.264
Hist. sal. × CG sal.	0.010 ± 0.153	37.144	0.067	0.947
**Hist. sal. × Comp.**	-2.535 ± 1.094	40.828	-2.318	**0.026**
CG sal. × Comp.	0.085 ± 0.314	1318.951	0.271	0.787
Hist. sal. × CG sal. × Comp.	0.246 ± 0.543	1212.736	0.453	0.651
R^2_{fixed}=0.0677, R^2_{total}=0.3131				

Table S4-b: CONTINUE TABLE S4

Dispersal ability	$\beta \pm$ SE	df	t-value	p-value
Hist. sal.	2.708 ± 42.385	16.880	0.064	0.950
CG sal.	1.682 ± 11.478	45.147	0.147	0.884
Comp.	-12.814 ± 72.470	95.401	-0.177	0.860
Biofrac. *P. aurelia*	64.084 ± 58.673	360.126	1.092	0.275
Density	1.485 ± 1.541	694.772	0.964	0.336
Hist. sal. × CG sal.	9.950 ± 15.473	34.450	0.643	0.524
Hist. sal. × Comp.	-113.832 ± 88.075	59.904	-1.292	0.201
CG sal. × Comp.	15.753 ± 31.286	1241.237	0.504	0.615
Hist. sal. × CG sal. × Comp.	19.026 ± 53.545	1090.019	0.355	0.722
R^2_{fixed}=0.0139, R^2_{total}=0.1427				

Table S5-a: Summary regression analysis for the partitioning of (ancestral) plasticity, mean trait evolution and evolution of plasticity of temporal trait change in *Paramecium aurelia* between the ancestral population of the selection phase (day 4) and each selected population evolved in the absence of competing species (given by historical salinity, i.e. the salinity used during the selection phase). Salinity gives the plasticity response to the salt concentration used in the common garden (given by the columns). Time reflects genetic trait change between the ancestral and selected population in the common garden (CG) salt concentration environment 0.5 g/l. Interaction between salinity and time reflect evolution of phenotypic plasticity between the ancestral and selected population. Density reflects intraspecific density of the species. Regression slope (β) $\pm$ standard error (SE), degrees of freedom (df), test statistic (t-value) and p-value are given for biomass (quantified as bio-area), cell shape (quantified as aspect ratio), and dispersal ability (quantified as gross speed). Significant effects ($p < 0.05$) are highlighted in bold.

Hist. sal.		CG salinity 1 g/l		CG salinity 2 g/l	
	Biomass	$\beta \pm$ SE	p-value	$\beta \pm$ SE	p-value
0	Salinity	-241.06 ± 156.58	0.162	-62.64055 ± 222.7373	0.7832
	Time	-1620.63 ± 192.85	< **0.001**	-1802.435 ± 334.2759	< **0.001**
	Sal × Time	363.37 ± 195.49	0.096	301.3565 ± 302.8968	0.341
	Density	12.91 ± 4.30	**0.015**	9.294269 ± 8.390000	0.292
0.5	Salinity	-183.46 ± 223.15	0.433	-146.24482 ± 247.9071	0.567
	Time	-1749.8741 ± 317.6903	< **0.001**	-1872.331 ± 442.4827	**0.001**
	Sal × Time	235.0883 ± 277.8622	0.418	284.4757 ± 301.9861	0.365
	Density	7.46468 ± 7.574856	0.351	3.812483 ± 11.428733	0.745
1	Salinity	-369.2948 ± 321.9744	0.277	-236.35233 ± 301.8192	0.460
	Time	-1119.1297 ± 514.1199	0.053	-1138.969 ± 628.6511	0.110
	Sal × Time	539.7907 ± 451.4140	0.260	316.0681 ± 378.7379	0.427
	Density	15.82679 ± 11.530099	0.202	15.887997 ± 14.273434	0.302
2	Salinity	-222.9073 ± 256.8702	0.448	-156.98655 ± 432.6351	0.726
	Time	-976.5064 ± 452.5327	0.099	-1257.833 ± 932.2956	0.212
	Sal × Time	462.9180 ± 385.8129	0.271	484.6132 ± 549.4663	0.399
	Density	10.91939 ± 8.913830	0.308	5.109899 ± 20.469520	0.809
4	Salinity	-181.1905 ± 192.6860	0.370	-201.78841 ± 198.0149	0.338
	Time	-1013.3876 ± 295.5224	**0.006**	-1202.540 ± 399.7508	**0.014**
	Sal × Time	-92.5375 ± 256.3023	0.725	348.7417 ± 259.3491	0.206
	Density	14.50652 ± 6.681975	0.061	11.768565 ± 9.384693	0.242

Table S5-b: CONTINUE TABLE S5.

Hist. sal.		CG salinity 1 g/l		CG salinity 2 g/l	
	Cell shape	$\beta \pm$ SE	p-value	$\beta \pm$ SE	p-value
0	Salinity	0.11 ± 0.10	0.317	0.03 ± 0.10	0.769
	Time	0.39 ± 0.12	**0.008**	0.25 ± 0.14	0.102
	Sal × Time	-0.17 ± 0.13	0.196	0.02 ± 0.13	0.854
	Density	-5.69e-03 ± 2.76e-03	0.062	-1.02e-02 ± 3.59e-03	**0.014**
0.5	Salinity	0.12 ± 0.18	0.512	0.05 ± 0.15	0.731
	Time	0.50 ± 0.25	0.072	0.39 ± 0.26	0.155
	Sal × Time	-0.04 ± 0.22	0.846	-0.31 ± 0.18	0.109
	Density	-6.97e-03 ± 6.10e-03	0.276	-1.01e-02 ± 6.70e-03	0.158
1	Salinity	0.12 ± 0.16	0.472	5.50e-03 ± 1.74e-01	0.975
	Time	0.45 ± 0.26	0.109	0.53 ± 0.36	0.171
	Sal × Time	-0.28 ± 0.23	0.262	-0.28 ± 0.23	0.262
	Density	-5.76e-03 ± 5.99e-03	0.356	-3.98e-03 ± 8.16e-03	0.637
2	Salinity	0.12 ± 0.22	0.608	0.01 ± 0.24	0.953
	Time	0.47 ± 0.38	0.246	0.54 ± 0.53	0.335
	Sal × Time	-0.30 ± 0.28	0.309	-0.54 ± 0.30	0.105
	Density	-6.90e-03 ± 7.84e-03	0.406	-5.25e-03 ± 1.17e-02	0.664
4	Salinity	0.11 ± 0.10	0.287	0.03 ± 0.11	0.795
	Time	0.47 ± 0.15	**0.010**	0.46 ± 0.21	0.054
	Sal × Time	-0.12 ± 0.13	0.395	-0.14 ± 0.13	0.308
	Density	-6.60e-03 ± 3.58e-03	0.096	-7.01e-03 ± 4.98e-03	0.189

Table S5-c: CONTINUE TABLE S5.

Hist. sal.		CG salinity 1 g/l		CG salinity 2 g/l	
	Dispersal ability	$\beta \pm$ SE	*p*-value	$\beta \pm$ SE	*p*-value
0	Salinity	135.54 ± 158.81	0.412	72.94 ± 114.80	0.536
	Time	28.39 ± 190.91	0.884	-126.33 ± 173.90	0.482
	Sal × Time	-255.24 ± 192.41	0.211	-178.53 ± 157.41	0.279
	Density	-13.66 ± 4.23	**0.008**	-18.23 ± 4.36	**0.001**
0.5	Salinity	72.12 ± 150.89	0.642	-12.00 ± 130.73	0.928
	Time	182.42 ± 213.58	0.410	157.42 ± 231.9	0.510
	Sal × Time	10.48 ± 184.85	0.956	-278.37 ± 158.42	0.104
	Density	-8.05 ± 5.13	0.143	-8.78 ± 6.00	0.169
1	Salinity	-43.11 ± 158.46	0.790	-2.82 ± 171.10	0.987
	Time	478.87 ± 253.81	0.082	241.75 ± 350.82	0.506
	Sal × Time	-156.17 ± 227.09	0.505	-156.17 ± 227.09	0.505
	Density	-4.72 ± 5.803	0.432	-10.02 ± 8.02	0.239
2	Salinity	72.69 ± 112.12	0.555	-22.94 ± 149.08	0.882
	Time	508.44 ± 189.48	0.055	541.91 ± 318.95	0.134
	Sal × Time	-193.82 ± 146.96	0.245	-470.55 ± 185.42	**0.036**
	Density	-8.11 ± 3.91	0.113	-7.36 ± 7.03	0.332
4	Salinity	91.839 ± 119.19	0.458	-40.45 ± 142.02	0.782
	Time	465.80 ± 181.23	**0.027**	399.58 ± 278.26	0.181
	Sal × Time	-253.25 ± 157.49	0.138	-46.37 ± 174.77	0.796
	Density	-4.85 ± 4.31	0.289	-5.02 ± 6.59	0.464

Table S6-a: Summary regression analysis for the partitioning of (ancestral) plasticity, mean trait evolution and evolution of plasticity of temporal trait change in *Paramecium aurelia* between the ancestral population of the selection phase (day 4) and each selected population evolved in the presence of competing species (given by historical salinity, i.e. the salinity used during the selection phase). Salinity gives the plasticity response to the salt concentration used in the common garden (given by the columns). Time reflects genetic trait change between the ancestral and selected population in the common garden (CG) salt concentration environment 0.5 g/l. Interaction between salinity and time reflect evolution of phenotypic plasticity between the ancestral and selected population. Density reflects intraspecific density of the species. Regression slope (β) $\pm$ standard error (SE), degrees of freedom (df), test statistic (t-value) and p-value are given for biomass (quantified as bio-area), cell shape (quantified as aspect ratio), and dispersal ability (quantified as gross speed). Significant effects ($p < 0.05$) are highlighted in bold.

Hist. sal.		CG salinity 1 g/l		CG salinity 2 g/l	
	Biomass	$\beta \pm$ SE	p-value	$\beta \pm$ SE	p-value
0	Salinity	-289.26 ± 325.57	0.395	-242.77 ± 279.70	0.406
	Time	-948.71 ± 414.39	**0.044**	-930.59 ± 388.68	**0.037**
	Sal × Time	-221.60 ± 396.35	0.588	-236.55 ± 334.89	0.496
	Density	16.32 ± 9.89	0.129	16.75 ± 9.64	0.112
0.5	Salinity	-218.20 ± 169.26	0.235	-159.61 ± 205.89	0.457
	Time	-1572.16 ± 244.65	< **0.001**	-1755.29 ± 332.92	< **0.001**
	Sal × Time	317.96 ± 208.23	0.160	474.88 ± 251.19	0.085
	Density	11.07 ± 5.52	0.081	5.79 ± 7.91	0.480
1	Salinity	-195.17 ± 284.61	0.509	-137.98 ± 240.43	0.580
	Time	-1217.66 ± 398.11	**0.012**	-1404.88 ± 360.53	**0.003**
	Sal × Time	129.51 ± 344.74	0.714	129.51 ± 344.74	0.714
	Density	8.24 ± 9.33	0.398	2.74 ± 8.55	0.755
2	Salinity	-198.11 ± 251.64	0.448	-191.04 ± 186.59	0.330
	Time	-1367.81 ± 318.34	**0.001**	-1307.21 ± 310.18	**0.001**
	Sal × Time	75.92 ± 301.04	0.805	752.47 ± 237.40	**0.008**
	Density	8.61 ± 7.59	0.280	10.37 ± 8.43	0.243
4	Salinity	-205.07 ± 155.49	0.227	-182.73 ± 187.12	0.358
	Time	-1854.25 ± 259.76	< **0.001**	-1896.99 ± 393.09	< **0.001**
	Sal × Time	251.42 ± 204.80	0.244	344.83 ± 241.64	0.181
	Density	10.33 ± 5.27	0.092	9.22 ± 8.79	0.323

Table S6-b: CONTINUE TABLE S6.

Hist. sal.		CG salinity 1 g/l		CG salinity 2 g/l	
	Cell shape	$\beta \pm$ SE	*p*-value	$\beta \pm$ SE	*p*-value
0	Salinity	0.09 ± 0.16	0.591	-4.73e-03 ± 1.40e-01	0.974
	Time	-0.05 ± 0.20	0.823	1.90e-04 ± 0.19	0.999
	Sal × Time	0.11 ± 0.19	0.586	0.29 ± 0.17	0.110
	Density	-4.04e-03 ± 4.84e-03	0.419	-2.59e-03 ± 4.81e-03	0.599
0.5	Salinity	0.13 ± 0.12	0.293	0.03 ± 0.12	0.810
	Time	0.15 ± 0.17	0.403	0.18 ± 0.20	0.394
	Sal × Time	-0.16 ± 0.14	0.274	-0.08 ± 0.15	0.621
	Density	-7.97e-03 ± 3.91e-03	0.068	-7.28e-03 ± 4.72e-03	0.150
1	Salinity	0.10 ± 0.15	0.526	0.03 ± 0.13	0.791
	Time	0.11 ± 0.20	0.601	2.99e-03 ± 0.19	0.988
	Sal × Time	-0.08 ± 0.18	0.667	-0.08 ± 0.18	0.667
	Density	-4.73e-03 ± 4.80e-03	0.344	-7.84e-03 ± 4.48e-03	0.105
2	Salinity	0.10 ± 0.12	0.426	0.02 ± 0.11	0.824
	Time	0.29 ± 0.15	0.068	0.24 ± 0.17	0.196
	Sal × Time	-0.13 ± 0.14	0.356	-0.19 ± 0.13	0.188
	Density	-4.72e-03 ± 3.50e-03	0.202	-6.45e-03 ± 4.75e-03	0.198
4	Salinity	0.11 ± 0.21	0.597	8.80e-03 ± 0.17	0.959
	Time	0.60 ± 0.33	0.097	0.67 ± 0.33	0.071
	Sal × Time	-0.24 ± 0.25	0.357	-0.13 ± 0.20	0.532
	Density	-6.30e-03 ± 7.12e-03	0.395	-4.42e-03 ± 7.59-e03	0.573
	Dispersal ability	$\beta \pm$ SE	*p*-value	$\beta \pm$ SE	*p*-value
0	Salinity	54.27 ± 140.42	0.706	-50.98 ± 144.12	0.730
	Time	-17.28 ± 178.49	0.925	74.43 ± 199.56	0.716
	Sal × Time	-20.30 ± 170.23	0.907	130.63 ± 171.65	0.461
	Density	-6.49 ± 4.25	0.153	-3.63 ± 4.94	0.476
0.5	Salinity	109.84 ± 146.64	0.471	8.29 ± 169.17	0.962
	Time	109.29 ± 207.36	0.610	110.71 ± 268.30	0.688
	Sal × Time	-178.24 ± 172.84	0.326	-133.22 ± 200.58	0.520
	Density	-11.39 ± 4.77	**0.038**	-11.48 ± 6.42	0.101
1	Salinity	93.90 ± 125.44	0.471	0.78 ± 160.678	0.996
	Time	69.04 ± 175.15	0.702	56.94 ± 237.10	0.815
	Sal × Time	-98.92 ± 150.93	0.526	-98.92 ± 150.93	0.526
	Density	-9.95 ± 4.11	**0.036**	-10.49 ± 5.62	0.088
2	Salinity	72.30 ± 110.81	0.528	-41.88 ± 78.27	0.607
	Time	154.05 ± 139.80	0.294	274.15 ± 129.70	0.0630
	Sal × Time	-157.60 ± 132.08	0.258	17.50 ± 99.31	0.864
	Density	-8.04 ± 3.34	**0.035**	-4.55 ± 3.53	0.228
4	Salinity	75.69 ± 92.08	0.434	-18.70 ± 55.95	0.750
	Time	482.95 ± 148.91	**0.010**	502.62 ± 121.40	**0.003**
	Sal × Time	-305.16 ± 113.57	**0.022**	-242.79 ± 75.84	**0.010**
	Density	-8.28 ± 3.14	**0.029**	-7.66 ± 2.67	**0.025**

Table S7-a: Summary regression analysis for the partitioning of (ancestral) plasticity, mean trait evolution and evolution of plasticity of temporal trait change in *Spirostomum teres* between the ancestral population of the selection phase (day 4) and each selected population evolved in the absence of competing species (given by historical salinity, i.e. the salinity used during the selection phase). Salinity gives the plasticity response to the salt concentration used in the common garden (given by the columns). Time reflects genetic trait change between the ancestral and selected population in the common garden (CG) salt concentration environment 0.5 g/l. Interaction between salinity and time reflect evolution of phenotypic plasticity between the ancestral and selected population. Density reflects intraspecific density of the species. Regression slope (β) $\pm$ standard error (SE), degrees of freedom (df), test statistic (t-value) and p-value are given for biomass (quantified as bio-area), cell shape (quantified as aspect ratio), and dispersal ability (quantified as gross speed). Significant effects ($p < 0.05$) are highlighted in bold.

Hist. sal.		CG salinity 1 g/l		CG salinity 2 g/l	
	Biomass	$\beta \pm$ SE	p-value	$\beta \pm$ SE	p-value
0	Salinity	835.89 ± 741.38	0.279	1607.80 ± 799.60	0.070
	Time	1559.84 ± 745.73	**0.049**	2237.93 ± 872.10	**0.023**
	Sal × Time	-292.33 ± 1017.07	0.779	-1537.39 ± 1025.80	0.159
	Density	-39.66 ± 30.14	0.216	-21.06 ± 33.13	0.539
0.5	Salinity	1333.30 ± 916.04	0.168	1603.51 ± 973.12	0.125
	Time	1850.07 ± 867.08	**0.050**	1754.87 ± 950.20	0.088
	Sal × Time	-1053.61 ± 1115.65	0.361	-1775.30 ± 1199.75	0.165
	Density	-21.91 ± 35.46	0.548	-30.95 ± 40.52	0.460
1	Salinity	1235.72 ± 1034.69	0.252	1604.30 ± 804.76	0.067
	Time	1625.88 ± 963.06	0.113	1818.88 ± 781.22	**0.034**
	Sal × Time	-579.29 ± 1249.50	0.650	-747.49 ± 983.72	0.460
	Density	-42.80375 ± 40.18151	0.306	-21.57503 ± 32.26167	0.516
2	Salinity	1362.90 ± 766.80	0.108	1571.81 ± 761.63	0.069
	Time	1723.06 ± 771.42	**0.049**	1512.59 ± 796.95	0.085
	Sal × Time	-885.15 ± 965.82	0.382	-717.63 ± 980.73	0.482
	Density	-15.80 ± 29.98	0.612	-33.01 ± 31.09	0.316
4	Salinity	1299.49 ± 327.93	< **0.001**	1592.31 ± 737.58	0.110
	Time	561.30 ± 648.84	0.388	401.01 ± 1253.06	0.763
	Sal × Time	-2386.09 ± 819.32	**0.004**	-507.11 ± 1595.26	0.760
	Density	-18.60 ± 11.79	0.116	-28.17 ± 31.41	0.433

Table S7-b: CONTINUE TABLE S7.

Hist. sal.		CG salinity 1 g/l		CG salinity 2 g/l	
	Cell shape	$\beta \pm$ SE	p-value	$\beta \pm$ SE	p-value
0	Salinity	-0.04 ± 13.17	0.901	-0.09 ± 6.80	0.705
	Time	-0.67 ± 0.36	0.074	-0.70 ± 0.29	**0.031**
	Sal × Time	-0.06 ± 0.46	0.905	0.22 ± 0.32	0.512
	Density	0.01 ± 0.01	0.393	7.93e-03 ± 9.44e-03	0.434
0.5	Salinity	-0.10 ± 13.47	0.892	-0.12 ± 12.64	0.868
	Time	0.59 ± 0.68	0.400	0.65 ± 0.70	0.367
	Sal × Time	0.07 ± 0.88	0.939	-0.17 ± 0.88	0.855
	Density	6.17e-03 ± 2.81e-02	0.830	0.01 ± 0.03	0.698
1	Salinity	-0.09 ± 13.18	0.858	-0.11 ± 12.87	0.822
	Time	0.09 ± 0.48	0.848	0.19 ± 0.45	0.673
	Sal × Time	-0.26 ± 0.62	0.683	0.27 ± 0.56	0.641
	Density	6.28e-03 ± 1.98e-02	0.757	0.02 ± 0.02	0.438
2	Salinity	-0.05 ± 11.49	0.922	-0.11 ± 9.86	0.865
	Time	0.09 ± 0.48	0.858	0.10 ± 0.66	0.879
	Sal × Time	-0.39 ± 0.60	0.530	0.09 ± 0.82	0.917
	Density	0.01 ± 0.02	0.454	0.02 ± 0.03	0.468
4	Salinity	-0.06 ± 2.52	0.881	-0.11 ± 203.00	0.591
	Time	1.701 ± 0.61	**0.049**	1.64 ± 0.46	< **0.001**
	Sal × Time	-2.71 ± 0.76	**0.020**	-2.05 ± 0.71	**0.004**
	Density	7.11e-03 ± 1.37e-02	0.658	3.51e-03 ± 8.33e-03	0.674
	Dispersal ability	$\beta \pm$ SE	p-value	$\beta \pm$ SE	p-value
0	Salinity	-9.22 ± 35.11	0.798	31.56 ± 35.38	0.393
	Time	71.66 ± 34.71	0.054	96.77 ± 38.57	< **0.001**
	Sal × Time	32.42 ± 48.54	0.520	-1.96 ± 45.37	0.966
	Density	-0.75 ± 1.44	0.615	0.19 ± 1.47	0.898
0.5	Salinity	5.18 ± 37.84	0.893	30.67 ± 48.55	0.539
	Time	181.81 ± 36.21	< **0.001**	182.19 ± 47.57	< **0.001**
	Sal × Time	-15.94 ± 45.86	0.733	-74.56 ± 59.85	0.235
	Density	-0.20 ± 1.43	0.894	-0.11 ± 2.02	0.956
1	Salinity	0.97 ± 37.24	0.980	33.88 ± 30.32	0.285
	Time	105.53 ± 34.87	**0.008**	122.39 ± 30.03	< **0.001**
	Sal × Time	3.43 ± 44.84	0.940	0.24 ± 36.98	0.995
	Density	-0.91 ± 1.42	0.535	0.54 ± 1.20	0.664
2	Salinity	6.56 ± 40.81	0.876	30.83 ± 39.80	0.458
	Time	147.37 ± 40.93	**0.004**	143.79 ± 41.30	**0.005**
	Sal × Time	-38.97 ± 51.41367	0.466	-44.52 ± 51.16	0.405
	Density	0.15 ± 1.60	0.929	-0.15 ± 1.63	0.930
4	Salinity	6.58 ± 12.55	0.600	32.26 ± 16.33	0.123
	Time	83.96 ± 24.83	< **0.001**	84.35 ± 31.77	**0.027**
	Sal × Time	-55.74 ± 31.35	0.077	-20.83 ± 46.09	0.656
	Density	-0.27 ± 0.45	0.543	-0.23 ± 0.67	0.748

Table S8-a: Summary regression analysis for the partitioning of (ancestral) plasticity, mean trait evolution and evolution of plasticity of temporal trait change in *Spirostomum teres* between the ancestral population of the selection phase (day 4) and each selected population evolved in the presence of competing species (given by historical salinity, i.e. the salinity used during the selection phase). Salinity gives the plasticity response to the salt concentration used in the common garden (given by the columns). Time reflects genetic trait change between the ancestral and selected population in the common garden (CG) salt concentration environment 0.5 g/l. Interaction between salinity and time reflect evolution of phenotypic plasticity between the ancestral and selected population. Density reflects intraspecific density of the species. Regression slope (β) $\pm$ standard error (SE), degrees of freedom (df), test statistic (t-value) and p-value are given for biomass (quantified as bio-area), cell shape (quantified as aspect ratio), and dispersal ability (quantified as gross speed). Significant effects ($p < 0.05$) are highlighted in bold.

Hist. sal.		CG salinity 1 g/l		CG salinity 2 g/l	
	Biomass	$\beta \pm$ SE	p-value	$\beta \pm$ SE	p-value
0	Salinity	1297.25 ± 340.95	< **0.001**	1593.73 ± 786.76	0.139
	Time	1203.97 ± 742.82	0.107	1154.38 ± 1166.28	0.358
	Sal × Time	601.41 ± 1174.61	0.609	-2010.34 ± 1475.91	0.206
	Density	-18.76 ± 12.25	0.128	-29.15 ± 33.55	0.455
0.5	Salinity	1336.45 ± 1044.22	0.241	1593.88 ± 694.81	0.072
	Time	-668.54 ± 1333.89	0.627	-696.15 ± 990.36	0.499
	Sal × Time	-1458.95 ± 1479.74	0.348	-563.55 ± 1158.66	0.638
	Density	-21.55 ± 42.82	0.631	-25.61 ± 29.30	0.426
1	Salinity	1298.83 ± 337.98	< **0.001**	1577.13 ± 664.33	0.069
	Time	-1020.41 ± 711.72	0.153	-1203.23 ± 1183.01	0.342
	Sal × Time	-261.74 ± 918.48	0.776	-908.58 ± 1319.43	0.512
	Density	-18.65 ± 12.15	0.126	-28.97 ± 28.14	0.360
	Cell shape	$\beta \pm$ SE	p-value	$\beta \pm$ SE	p-value
0	Salinity	-0.05 ± 2.79	0.897	-0.11 ± 201.00	0.612
	Time	0.63 ± 0.60	0.313	0.62 ± 0.52	0.233
	Sal × Time	0.15 ± 0.91	0.873	0.16 ± 0.72	0.821
	Density	7.51e-03 ± 1.34e-02	0.629	4.00e-03 ± 8.50e-03	0.651
0.5	Salinity	-0.09 ± 5.63	0.900	-0.12 ± 1.62	0.822
	Time	-0.69 ± 0.90	0.467	-0.57 ± 0.65	0.439
	Sal × Time	-0.31 ± 1.00	0.768	0.35 ± 0.77	0.678
	Density	7.43e-03 ± 2.91e-03	0.808	8.54e-03 ± 1.90e-02	0.709
1	Salinity	-0.06 ± 2.85	0.882	-0.11 ± 213.00	0.604
	Time	-1.29 ± 0.63	0.089	-1.35 ± 0.50	**0.007**
	Sal × Time	0.82 ± 0.78	0.324	0.85 ± 0.56	0.130
	Density	6.87e-03 ± 1.34e-03	0.656	3.67e-03 ± 8.48e-03	0.666

Table S8-b: CONTINUE TABLE S8

Hist. sal.		CG salinity 1 g/l		CG salinity 2 g/l	
	Dispersal ability	β ± SE	p-value	β ± SE	p-value
0	Salinity	4.33 ± 90.0	0.964	30.31 ± 87.62	0.750
	Time	150.72 ± 108.36	0.238	154.66 ± 108.73	0.230
	Sal × Time	150.93 ± 137.86	0.330	-7.96 ± 127.94	0.953
	Density	-0.14 ± 3.76	0.97	0.089 ± 3.78	0.983
0.5	Salinity	3.38 ± 63.81	0.962	29.98 ± 87.29	0.748
	Time	192.86 ± 77.42	0.097	203.70 ± 104.94	0.111
	Sal × Time	-108.70 ± 86.64	0.310	-94.05 ± 120.98	0.472
	Density	-0.40 ± 2.64	0.893	-0.51 ± 3.73	0.898
1	Salinity	6.66 ± 12.64	0.599	30.16 ± 61.95	0.654
	Time	79.50 ± 26.62	**0.003**	77.54 ± 100.81	0.484
	Sal × Time	-35.79 ± 34.35	0.299	63.14 ± 110.86	0.598
	Density	-0.27 ± 0.45	0.554	-0.30 ± 2.68	0.915

Table S9: Summary of regression analysis for the phenotypic plasticity response to salinity of *Paramecium aurelia* evolved in the absence of competing species. Salinity gives the plasticity response to the salt concentration used in the common garden (given by the columns). Density reflects intraspecific density of the species. Regression slope (β) ± standard error (SE), degrees of freedom (df), test statistic (t-value) and p-value are given for biomass (quantified as bio-area), cell shape (quantified as aspect ratio), and dispersal ability (quantified as gross speed). Significant effects ($p < 0.05$) are highlighted in bold.

Hist. sal.		CG salinity 1 g/l		CG salinity 2 g/l		CG salinity 4 g/l	
	Biomass	β ± SE	p-value	β ± SE	p-value	β ± SE	p-value
Anc.	Salinity	-209.17 ± 189.06	0.343	-196.89 ± 230.89	0.457	NA ± NA	NA
	Density	10.49 ± 6.51	0.204	11.01 ± 11.20	0.394	NA ± NA	NA
0	Salinity	114.80 ± 51.36	**0.026**	221.02 ± 58.95	< **0.001**	NA ± NA	NA
	Density	6.71 ± 5.11	0.193	7.92 ± 3.12	**0.031**	NA ± NA	NA
0.5	Salinity	-57.16 ± 62.55	0.361	91.60 ± 56.93	0.108	NA ± NA	NA
	Density	-38.98 ± 12.60	**0.002**	46.38 ± 32.88	0.167	NA ± NA	NA
1	Salinity	256.21 ± 138.41	0.065	245.66 ± 136.72	0.075	1645.87 ± 388.36	< **0.001**
	Density	66.71 ± 77.48	0.390	-46.56 ± 63.98	0.470	333.40 ± 124.59	**0.030**
2	Salinity	327.04 ± 213.28	0.131	582.67 ± 183.84	**0.002**	383.58 ± 229.41	0.098
	Density	357.65 ± 247.78	0.166	-129.18 ± 63.77	0.051	-16.89 ± 132.36	0.899
4	Salinity	-390.23 ± 90.74	< **0.001**	113.08 ± 88.38	0.202	238.35 ± 88.15	**0.007**
	Density	64.71 ± 12.91	< **0.001**	-1.89 ± 21.04	0.929	4.78 ± 17.84	0.789

Table S9-b: CONTINUE TABLE S9.

Hist. sal.		CG salinity 1 g/l		CG salinity 2 g/l		CG salinity 4 g/l	
	Cell shape	$\beta \pm$ SE	p-value	$\beta \pm$ SE	p-value	$\beta \pm$ SE	p-value
Anc.	Salinity	0.12 ± 0.12	0.415	0.03 ± 0.12	0.811	NA ± NA	NA
	Density	-6.49e-03 ± 4.30e-03	0.227	7.47e-03 ± 5.85e-03	0.290	NA ± NA	NA
0	Salinity	-0.09 ± 0.04	**0.045**	4.44e-03 ± 4.35e-02	0.919	NA ± NA	NA
	Density	4.72e-03 ± 5.11e-03	0.383	-7.17e-03 ± 5.60e-03	0.233	NA ± NA	NA
0.5	Salinity	0.08 ± 0.05	0.115	-0.28 ± 0.05	< **0.001**	NA ± NA	NA
	Density	-8.09e-03 ± 9.99e-03	0.418	-0.08 ± 0.02	**0.005**	NA ± NA	NA
1	Salinity	-0.22 ± 0.09	**0.020**	-0.24 ± 0.09	0.014	-0.71 ± 0.26	**0.009**
	Density	0.08 ± 0.05	0.129	0.06 ± 0.04	0.105	0.16 ± 0.07	0.06
2	Salinity	-0.39 ± 0.13	**0.003**	-0.47 ± 0.11	< **0.001**	-0.34 ± 0.15	**0.025**
	Density	-0.29 ± 0.14	0.054	-0.04 ± 0.04	0.380	-0.09 ± 0.08	0.276
4	Salinity	0.02 ± 0.06	0.779	-0.09 ± 0.06	0.140	-0.50 ± 0.05	< **0.001**
	Density	5.69e-04 ± 0.01	0.967	-0.02 ± 0.01	**0.015**	7.46e-04 ± 8.97e-03	0.935
	Dispersal ability	$\beta \pm$ SE	p-value	$\beta \pm$ SE	p-value	$\beta \pm$ SE	p-value
Anc.	Salinity	69.95 ± 66.94	0.371	-26.03 ± 65.11	0.716	NA ± NA	NA
	Density	-7.82 ± 2.33	**0.044**	-6.88 ± 3.13	0.113	NA ± NA	NA
0	Salinity	-125.29 ± 44.58	**0.005**	-46.28 ± 41.45	0.265	NA ± NA	NA
	Density	13.20 ± 6.04	**0.037**	-24.19 ± 4.70	< **0.001**	NA ± NA	NA
0.5	Salinity	48.01 ± 50.82	0.345	-326.41 ± 42.47	< **0.001**	NA ± NA	NA
	Density	-22.02 ± 10.41	**0.035**	-34.54 ± 19.19	0.093	NA ± NA	NA
1	Salinity	85.23 ± 95.53	0.374	-91.78 ± 84.23	0.280	-368.40 ± 298.43	0.224
	Density	-5.08 ± 53.18	0.924	-47.10 ± 34.20	0.188	-46.88 ± 106.38	0.673
2	Salinity	-196.43 ± 98.36	**0.049**	-425.71 ± 103.07	< **0.001**	-556.01 ± 83.89	< **0.001**
	Density	-77.97 ± 81.02	0.339	-31.88 ± 34.52	0.366	-50.20 ± 50.42	0.329
4	Salinity	-219.34 ± 63.28	< **0.001**	-85.77 ± 62.29	0.170	-402.96 ± 49.64	< **0.001**
	Density	10.75 ± 9.82	0.280	4.88 ± 15.38	0.756	-9.85 ± 7.81	0.209

Table S10-a: Summary of regression analysis for the phenotypic plasticity response to salinity of *Paramecium aurelia* evolved in the presence of competing species. Salinity gives the plasticity response to the salt concentration used in the common garden (given by the columns). Density reflects intraspecific density of the species. Regression slope (β) $\pm$ standard error (SE), degrees of freedom (df), test statistic (t-value) and p-value are given for biomass (quantified as bio-area), cell shape (quantified as aspect ratio), and dispersal ability (quantified as gross speed). Significant effects ($p < 0.05$) are highlighted in bold.

Hist. sal.		CG salinity 1 g/l		CG salinity 2 g/l		CG salinity 4 g/l	
	Biomass	$\beta \pm$ SE	p-value	$\beta \pm$ SE	p-value	$\beta \pm$ SE	p-value
Anc.	Salinity	41.73 ± 100.32	0.678	66.28 ± 155.33	0.700	182.53 ± 292.05	0.704
	Density	-14.67 ± 3.55	< **0.001**	-14.19 ± 5.56	0.065	-13.66 ± 5.12	0.488
0	Salinity	-532.25 ± 71.05	< **0.001**	-521.42 ± 74.93	< **0.001**	NA ± NA	NA
	Density	24.36 ± 7.78	**0.002**	5.92 ± 5.67	0.303	NA ± NA	NA
0.5	Salinity	262.30 ± 89.13	**0.004**	371.18 ± 79.28	< **0.001**	NA ± NA	NA
	Density	-37.41 ± 16.67	**0.021**	-11.69 ± 6.50	0.075	NA ± NA	NA
1	Salinity	-114.39 ± 76.60	0.136	203.40 ± 82.49	0.014	686.69 ± 301.02	0.025
	Density	11.58 ± 12.84	0.369	-10.07 ± 5.91	0.091	12.14 ± 23.93	0.649
2	Salinity	-270.57 ± 76.94	< **0.001**	492.68 ± 78.56	< **0.001**	516.94 ± 84.43	< **0.001**
	Density	26.06 ± 7.44	< **0.001**	-8.23 ± 13.01	0.528	-4.59 ± 9.80	0.640
4	Salinity	16.94 ± 105.32	0.873	219.59 ± 113.71	0.054	398.24 ± 108.91	**0.001**
	Density	14.33 ± 18.43	0.459	-12.46 ± 18.78	0.508	-39.17 ± 23.74	0.164

Table S10-b: CONTINUE TABLE S10.

Hist. sal.		CG salinity 1 g/l		CG salinity 2 g/l		CG salinity 4 g/l	
	Cell shape	$\beta \pm$ SE	p-value	$\beta \pm$ SE	p-value	$\beta \pm$ SE	p-value
Anc.	Salinity	-0.02 ± 0.11	0.893	0.05 ± 0.03	0.093	-0.11 ± 0.19	0.613
	Density	2.14e-03 ± 3.96e-03	0.626	4.28e-04 ± 1.34e-03	0.751	6.64e-04 ± 3.64e-03	0.873
0	Salinity	0.18 ± 0.04	< **0.001**	0.32 ± 0.05	< **0.001**	NA ± NA	NA
	Density	2.03e-03 ± 4.30e-03	0.638	-9.00e-03 ± 4.21e-03	**0.034**	NA ± NA	NA
0.5	Salinity	-0.06 ± 0.06	0.248	-0.10 ± 0.05	**0.028**	NA ± NA	NA
	Density	-1.16e-03 ± 9.05e-03	0.650	7.11e-04 ± 3.83e-03	0.853	NA ± NA	NA
1	Salinity	0.03 ± 0.04	0.405	0.03 ± 0.04	0.412	-0.57 ± 0.16	< **0.001**
	Density	6.67e-03 ± 6.72e-03	0.324	-5.29e-03 ± 2.98e-03	0.077	-9.71e-03 ± 1.80e-02	0.615
2	Salinity	-0.04 ± 0.04	0.345	-0.15 ± 0.04	< **0.001**	-0.46 ± 0.05	< **0.001**
	Density	-5.58e-03 ± 3.78e-03	0.144	2.78e-03 ± 7.47e-03	0.710	2.33e-03 ± 5.29e-03	0.660
4	Salinity	-0.18 ± 0.09	**0.033**	-0.16 ± 0.08	0.835	-0.22 ± 0.08	**0.013**
	Density	0.02 ± 0.02	0.227	0.02 ± 0.01	0.507	-0.03 ± 0.02	0.236
	Dispersal ability	$\beta \pm$ SE	p-value	$\beta \pm$ SE	p-value	$\beta \pm$ SE	p-value
Anc.	Salinity	42.30 ± 93.07	0.680	-50.73 ± 45.65	0.348	-109.05 ± 51.07	**0.033**
	Density	1.48 ± 3.35	0.687	0.08 ± 1.58	0.961	0.53 ± 0.82	0.514
0	Salinity	10.12 ± 36.86	0.784	64.68 ± 42.51	0.129	NA ± NA	NA
	Density	0.50 ± 4.02	0.901	-2.51 ± 3.83	0.513	NA ± NA	NA
0.5	Salinity	30.49 ± 51.58	0.555	-121.90 ± 43.50	**0.005**	NA ± NA	NA
	Density	-27.18 ± 8.75	0.003	-12.10 ± 3.68	**0.001**	NA ± NA	NA
1	Salinity	45.74 ± 43.23	0.291	17.66 ± 47.30	0.710	-681.62 ± 178.18	< **0.001**
	Density	-19.17 ± 6.93	**0.007**	-5.65 ± 3.48	0.106	-36.63 ± 18.14	0.117
2	Salinity	-127.64 ± 37.76	< **0.001**	-28.12 ± 44.10	0.524	-480.26 ± 33.85	< **0.001**
	Density	-8.05 ± 3.33	**0.021**	3.31 ± 6.62	0.617	-1.90 ± 3.82	0.621
4	Salinity	-179.48 ± 89.49	**0.049**	-224.12 ± 75.99	**0.003**	-241.22 ± 67.45	**0.001**
	Density	-22.75 ± 17.34	0.210	-17.92 ± 10.42	0.087	-62.65 ± 12.27	**0.046**

Table S11-a: Summary of regression analysis for the phenotypic plasticity response to salinity of *Spirostomum teres* evolved in the absence of competing species. Salinity gives the plasticity response to the salt concentration used in the common garden (given by the columns). Density reflects intraspecific density of the species. Regression slope (β) $\pm$ standard error (SE), degrees of freedom (df), test statistic (t-value) and p-value are given for biomass (quantified as bio-area), cell shape (quantified as aspect ratio), and dispersal ability (quantified as gross speed). Significant effects ($p < 0.05$) are highlighted in bold.

Hist. sal.	Traits	CG salinity 1 g/l		CG salinity 2 g/l		CG salinity 4 g/l	
	Biomass	$\beta \pm$ SE	p-value	$\beta \pm$ SE	p-value	$\beta \pm$ SE	p-value
Anc.	Salinity	1299.63 ± 341.49	< **0.001**	1589.35 ± 750.41	0.121	NA ± NA	NA
	Density	-18.59 ± 12.27	0.132	-29.21 ± 31.97	0.431	NA ± NA	NA
0	Salinity	332.90 ± 354.42	0.349	34.14 ± 408.09	0.933	-932.05 ± 1339.19	0.493
	Density	-194.27 ± 128.41	0.133	41.78 ± 102.95	0.688	-459.47 ± 247.54	0.134
0.5	Salinity	146.86 ± 223.00	0.511	637.10 ± 311.48	**0.042**	1631.62 ± 771.44	**0.040**
	Density	22.78 ± 47.55	0.632	298.84 ± 70.81	< **0.001**	181.47 ± 122.97	0.150
1	Salinity	46.88 ± 265.36	0.860	1015.13 ± 281.91	< **0.001**	448.46 ± 503.69	0.375
	Density	-210.64 ± 41.51	< **0.001**	-41.92 ± 60.24	0.496	-184.08 ± 75.05	**0.019**
2	Salinity	1006.50 ± 413.22	**0.016**	758.17 ± 411.12	0.067	852.59 ± 424.71	**0.046**
	Density	134.87 ± 79.70	0.095	-101.01 ± 84.08	0.236	-31.64 ± 62.10	0.611
4	Salinity	-3780.74 ± 7186.62	1.000	1300.72 ± 2292.59	0.579	936.32 ± 1265.21	0.475
	Density	-5762.06 ± 11573.51	1.000	666.76 ± 1317.42	0.621	NA ± NA	NA

Table S11-b: TABLE S11 CONTINUED

Hist. sal.	Traits	CG salinity 1 g/l		CG salinity 2 g/l		CG salinity 4 g/l	
	Cell shape	$\beta \pm$ SE	*p*-value	$\beta \pm$ SE	*p*-value	$\beta \pm$ SE	*p*-value
Anc.	Salinity	-0.05 ± 0.34	0.894	-0.11 ± 0.22	0.604	NA ± NA	NA
	Density	6.93e-03 ± 1.30e-02	0.644	3.56e-03 ± 8.57e-03	0.679	NA ± NA	NA
0	Salinity	0.12 ± 0.142	0.369	0.21 ± 0.15	0.148	1.12 ± 0.42	**0.017**
	Density	0.18 ± 0.05	< **0.001**	0.09 ± 0.03	**0.006**	0.21 ± 0.07	0.030
0.5	Salinity	0.14 ± 0.15	0.338	-0.09 ± 0.22	0.672	0.17 ± 0.55	0.762
	Density	-0.13 ± 0.03	< **0.001**	0.11 ± 0.05	**0.033**	0.13 ± 0.09	0.135
1	Salinity	-0.64 ± 0.13	< **0.001**	0.19 ± 0.15	0.196	-0.29 ± 0.24	0.230
	Density	-0.03 ± 0.02	0.146	0.05 ± 0.04	0.186	0.02 ± 0.04	0.633
2	Salinity	-0.59 ± 0.18	< **0.001**	-0.30 ± 0.17	0.081	0.66 ± 0.30	**0.034**
	Density	4.55e-03 ± 3.45e-02	0.895	-0.15 ± 0.04	< **0.001**	0.26 ± 0.06	< **0.001**
4	Salinity	-7.88 ± 4.88	1.000	-2.20 ± 1.55	0.177	-2.08 ± 3.63	0.578
	Density	-10.71 ± 7.85	1.000	-0.155 ± 0.890	0.864	NA ± NA	NA
	Dispersal ability	$\beta \pm$ SE	*p*-value	$\beta \pm$ SE	*p*-value	$\beta \pm$ SE	*p*-value
Anc.	Salinity	6.61 ± 12.22	0.589	32.25 ± 16.65	0.135	NA ± NA	NA
	Density	-0.27 ± 0.44	0.535	-0.23 ± 0.68	0.760	NA ± NA	NA
0	Salinity	13.79 ± 17.23	0.425	38.23 ± 19.72	0.055	-19.89 ± 68.64	0.775
	Density	-2.27 ± 6.20	0.715	9.57 ± 5.07	0.067	-17.56 ± 14.19	0.265
0.5	Salinity	-7.16 ± 14.58	0.623	-14.78 ± 19.19	0.442	45.32 ± 48.09	0.349
	Density	0.43 ± 2.98	0.886	11.40 ± 4.35	**0.009**	14.11 ± 7.73	0.074
1	Salinity	-22.35 ± 13.61	0.102	47.30 ± 14.34	**0.001**	42.21 ± 21.30	0.049
	Density	-7.43 ± 2.06	< **0.001**	7.03 ± 3.03	0.032	-0.17 ± 2.27	0.941
2	Salinity	-7.19 ± 21.84	0.742	-14.71 ± 20.16	0.467	9.47 ± 33.69	0.781
	Density	5.14 ± 4.25	0.232	-1.24 ± 4.37	0.777	1.95 ± 6.42	0.766
4	Salinity	-511.84 ± 446.97	1.000	8.73 ± 36.30	0.813	78.19 ± 80.70	0.353
	Density	-986.63 ± 729.50	1.000	-11.18 ± 24.14	0.650	NA ± NA	NA

Table S12-a: Summary of regression analysis for the phenotypic plasticity response to salinity of *Spirostomum teres* evolved in the presence of competing species. Salinity gives the plasticity response to the salt concentration used in the common garden (given by the columns). Density reflects intraspecific density of the species. Regression slope (β) $\pm$ standard error (SE), degrees of freedom (df), test statistic (t-value) and p-value are given for biomass (quantified as bio-area), cell shape (quantified as aspect ratio), and dispersal ability (quantified as gross speed). Significant effects ($p < 0.05$) are highlighted in bold.

Hist. sal.	Traits	CG salinity 1 g/l		CG salinity 2 g/l		CG salinity 4 g/l	
	Biomass	$\beta \pm$ SE	p-value	$\beta \pm$ SE	p-value	$\beta \pm$ SE	p-value
Anc.	Salinity	857.67 ± 1081.13	0.490	881.74 ± 594.28	0.147	NA ± NA	NA
	Density	250.64 ± 249.12	0.373	132.81 ± 150.42	0.383	NA ± NA	NA
0	Salinity	3292.60 ± 814.65	**0.004**	733.12 ± 1713.16	0.686	NA ± NA	NA
	Density	253.64 ± 401.10	0.544	-159.87 ± 1182.46	0.900	NA ± NA	NA
0.5	Salinity	23.57 ± 696.48	0.973	866.56 ± 626.02	0.178	3007.56 ± 1379.42	0.051
	Density	-617.01 ± 284.15	0.071	62.46 ± 136.96	0.652	135.54 ± 784.23	0.875
1	Salinity	1261.89 ± 1497.84	0.412	1217.49 ± 1762.23	0.496	NA ± NA	NA
	Density	-717.43 ± 1607.49	0.661	466.69 ± 1490.24	0.757	NA ± NA	NA
	Cell shape	$\beta \pm$ SE	p-value	$\beta \pm$ SE	p-value	$\beta \pm$ SE	p-value
Anc.	Salinity	0.19 ± 0.32	0.555	0.16 ± 0.36	0.652	NA ± NA	NA
	Density	0.07 ± 0.08	0.388	3.29e-03 ± 9.05e-03	0.997	NA ± NA	NA
0	Salinity	1.09 ± 0.67	0.139	1.14 ± 0.75	0.174	NA ± NA	NA
	Density	0.55 ± 0.32	0.123	0.67 ± 0.42	0.214	NA ± NA	NA
0.5	Salinity	-0.76 ± 0.40	0.060	-0.20 ± 0.71	0.791	-0.17 ± 0.90	0.851
	Density	-0.27 ± 0.20	0.202	0.20 ± 0.19	0.372	-0.35 ± 0.50	0.551
1	Salinity	1.18 ± 1.28	0.372	2.03 ± 0.96	**0.045**	NA ± NA	NA
	Density	-1.24 ± 1.31	0.359	1.25 ± 0.81	0.137	NA ± NA	NA

Table S12-b: TABLE S12 CONTINUED

Hist. sal.	Traits	CG salinity 1 g/l		CG salinity 2 g/l		CG salinity 4 g/l	
	Dispersal ability	$\beta \pm$ SE	p-value	$\beta \pm$ SE	p-value	$\beta \pm$ SE	p-value
Anc.	Salinity	-6.55 ± 33.95	0.848	22.04 ± 50.06	0.700	NA ± NA	NA
	Density	-9.68 ± 8.93	0.284	-3.85 ± 1.09	0.769	NA ± NA	NA
0	Salinity	271.97 ± 77.66	**0.007**	135.63 ± 116.15	0.289	NA ± NA	NA
	Density	16.56 ± 38.16	0.675	47.34 ± 71.22	0.558	NA ± NA	NA
0.5	Salinity	-69.45 ± 37.57	0.071	-70.45 ± 69.96	0.407	-1.93 ± 88.85	0.983
	Density	-2.65 ± 17.74	0.885	-4.65 ± 17.98	0.829	14.85 ± 84.86	0.874
1	Salinity	-43.87 ± 60.36	0.478	-42.11 ± 219.52	0.875	NA ± NA	NA
	Density	45.53 ± 75.56	0.555	-62.26 ± 275.06	0.858	NA ± NA	NA

Table S13-a: Genetic trait difference for high salinity selected *Paramecium aurelia* populations comparing those evolved in the absence and presence of competing species. Competition reflects genetic trait differences between the selected populations evolved in the absence and presence of competing species. Density reflects intraspecific density of the species. Calculations use trait values from the salinity common garden environment 0.5, 1, 2 and 4 g/l, respectively for the traits: biomass, cell shape and dispersal ability. The last column gives the summary statistics of the output when excluding microcosm ID 120 for which *S.teres* was found.

CG sal.	Traits	Historical salinity 2 g/l		Historical salinity 4 g/l		Historical salinity 4 g/l (without ID 120)	
	Biomass	$\beta \pm$ SE	p-value	$\beta \pm$ SE	p-value	$\beta \pm$ SE	p-value
0.5	Competition	-477.48 ± 419.53	0.293	-555.09 ± 125.89	**0.025**	-619.87 ± 96.53	< **0.001**
	Density	31.17 ± 27.37	0.309	32.49 ± 20.94	0.141	42.62 ± 18.65	**0.023**
1	Competition	-562.44 ± 422.10	0.230	-194.41 ± 157.29	0.249	-267.61 ± 182.14	0.188
	Density	4.28 ± 23.08	0.859	57.13 ± 22.68	**0.035**	61.38 ± 24.29	**0.043**
2	Competition	915.64 ± 428.06	0.065	-444.34 ± 270.81	0.174	-609.61 ± 261.44	0.097
	Density	-131.08 ± 47.80	0.065	-25.49 ± 19.85	0.200	-26.82 ± 19.68	0.174
4	Competition	124.87 ± 229.04	0.600	-503.52 ± 186.63	**0.027**	-538.74 ± 235.58	0.207
	Density	-15.34 ± 17.49	0.468	-5.18 ± 31.51	0.874	14.35 ± 35.56	0.700
	Cell shape	$\beta \pm$ SE	p-value	$\beta \pm$ SE	p-value	$\beta \pm$ SE	p-value
0.5	Competition	-0.289 ± 0.235	0.273	0.103 ± 0.153	0.534	0.059 ± 0.177	0.764
	Density	-0.003 ± 0.019	0.893	-0.009 ± 0.030	0.776	-0.015 ± 0.026	0.581
1	Competition	-0.170 ± 0.153	0.304	0.004 ± 0.127	0.970	0.035 ± 0.155	0.835
	Density	0.000 ± 0.005	0.968	-0.015 ± 0.018	0.425	-0.008 ± 0.014	0.600
2	Competition	-0.136 ± 0.235	0.579	0.055 ± 0.069	0.446	0.064 ± 0.080	0.454
	Density	0.027 ± 0.030	0.403	0.029 ± 0.012	0.074	0.028 ± 0.015	0.129
4	Competition	-0.274 ± 0.132	0.070	0.329 ± 0.119	**0.047**	0.353 ± 0.144	0.090
	Density	-0.005 ± 0.011	0.665	0.001 ± 0.011	0.952	0.001 ± 0.010	0.854

Table S13-b: TABLE S13 CONTINUED

	Dispersal ability	$\beta \pm$ SE	p-value	$\beta \pm$ SE	p-value	$\beta \pm$ SE	p-value
0.5	Competition	-529.62 $\pm$ 151.42	**0.006**	229.81 $\pm$ 91.27	0.088	189.89 $\pm$ 73.22	**0.010**
	Density	10.02 $\pm$ 11.68	0.421	10.03 $\pm$ 15.17	0.518	18.79 $\pm$ 14.15	0.186
1	Competition	-295.26 $\pm$ 144.90	0.087	176.77 $\pm$ 103.38	0.120	151.29 $\pm$ 112.42	0.216
	Density	-8.18 $\pm$ 3.66	**0.026**	4.24 $\pm$ 14.85	0.782	8.45 $\pm$ 14.95	0.588
2	Competition	153.93 $\pm$ 125.96	0.240	26.67 $\pm$ 136.68	0.854	-10.23 $\pm$ 172.98	0.956
	Density	-18.90 $\pm$ 15.10	0.212	-8.17 $\pm$ 14.36	0.665	-1.36 $\pm$ 15.11	0.942
4	Competition	-238.70 $\pm$ 97.31	0.073	207.96 $\pm$ 136.61	0.219	227.22 $\pm$ 184.51	0.318
	Density	-4.99 $\pm$ 4.00	0.390	-25.23 $\pm$ 12.63	0.122	-18.70 $\pm$ 10.12	0.127

Table S14-a: Trait difference due to historical competition and the presence of competitors for the salinity selected *Paramecium aurelia* populations evolved in the 0, 0.5 and 1 g/l salt conditions. Competition reflects trait differences between the selected populations evolved in the absence and presence of competing species. Density reflects intraspecific density of the species. Calculations use trait values from the salinity common garden environment 0.5, 1, 2 and 4 g/l, respectively for the traits: biomass, cell shape and dispersal ability.

CG sal.	Traits	Historical salinity 0 g/l		Historical salinity 0.5 g/l		Historical salinity 1 g/l	
	Biomass	$\beta \pm$ SE	*p*-value	$\beta \pm$ SE	*p*-value	$\beta \pm$ SE	*p*-value
0	Competition	302.03 ± 214.12	0.235	/	/	/	/
	Density	-5.78 ± 19.70	0.773	/	/	/	/
0.5	Competition	557.01 ± 261.53	0.144	27.73 ± 183.53	0.889	248.71 ± 356.92	0.520
	Density	32.59 ± 18.08	0.140	-16.87 ± 27.77	0.564	15.21 ± 28.32	0.614
1	Competition	-42.37 ± 213.05	0.853	97.09 ± 175.39	0.595	-231.50 ± 484.51	0.653
	Density	13.77 ± 10.89	0.241	7.89 ± 21.32	0.722	49.67 ± 30.71	0.212
2	Competition	32.27 ± 222.59	0.892	296.05 ± 200.27	0.207	572.27 ± 418.09	0.249
	Density	-2.76 ± 10.41	0.799	-10.25 ± 13.78	0.481	-15.31 ± 24.23	0.560
4	Competition	/	/	/	/	-8.94 ± 1152.50	0.994
	Density	/	/	/	/	388.36 ± 527.16	0.473
	Cell shape	$\beta \pm$ SE	*p*-value	$\beta \pm$ SE	*p*-value	$\beta \pm$ SE	*p*-value
0	Competition	-0.225 ± 0.136	0.178	/	/	/	/
	Density	-0.008 ± 0.012	0.533	/	/	/	/
0.5	Competition	-0.490 ± 0.131	**0.028**	-0.333 ± 0.118	**0.020**	-0.491 ± 0.170	**0.020**
	Density	-0.001 ± 0.008	0.865	-0.022 ± 0.018	0.245	0.014 ± 0.019	0.490
1	Competition	-0.219 ± 0.139	0.197	-0.426 ± 0.149	**0.036**	-0.308 ± 0.204	0.174
	Density	0.003 ± 0.006	0.644	-0.008 ± 0.016	0.633	0.013 ± 0.027	0.646
2	Competition	-0.234 ± 0.129	0.147	-0.085 ± 0.115	0.505	-0.162 ± 0.140	0.336
	Density	-0.006 ± 0.007	0.385	-0.011 ± 0.008	0.185	-0.010 ± 0.009	0.344
4	Competition	/	/	/	/	-0.172 ± 0.602	0.779
	Density	/	/	/	/	-0.191 ± 0.262	0.479

Table S14-b: TABLE S14 CONTINUED

	Dispersal ability	$\beta \pm$ SE	p-value	$\beta \pm$ SE	p-value	$\beta \pm$ SE	p-value
0	Competition	31.47 ± 121.60	0.809	/	/	/	/
	Density	-30.25 ± 12.35	**0.035**	/	/	/	/
0.5	Competition	-269.18 ± 146.30	0.163	-1.84 ± 159.91	0.991	-83.07 ± 190.02	0.672
	Density	-14.58 ± 6.21	0.060	-44.10 ± 18.45	**0.042**	-25.07 ± 21.16	0.270
1	Competition	-112.04 ± 160.49	0.540	-93.95 ± 127.97	0.496	78.07 ± 153.80	0.627
	Density	-5.12 ± 8.60	0.569	-13.74 ± 13.52	0.342	-23.96 ± 20.89	0.294
2	Competition	-20.69 ± 178.00	0.914	233.86 ± 135.19	0.193	9.90 ± 207.63	0.965
	Density	-10.82 ± 9.30	0.284	-17.51 ± 9.87	0.117	-17.36 ± 13.54	0.294
4	Competition	/	/	/	/	-92.03 ± 11121.00	0.936
	Density	/	/	/	/	-223.81 ± 477.72	0.647

Supplementary Figures

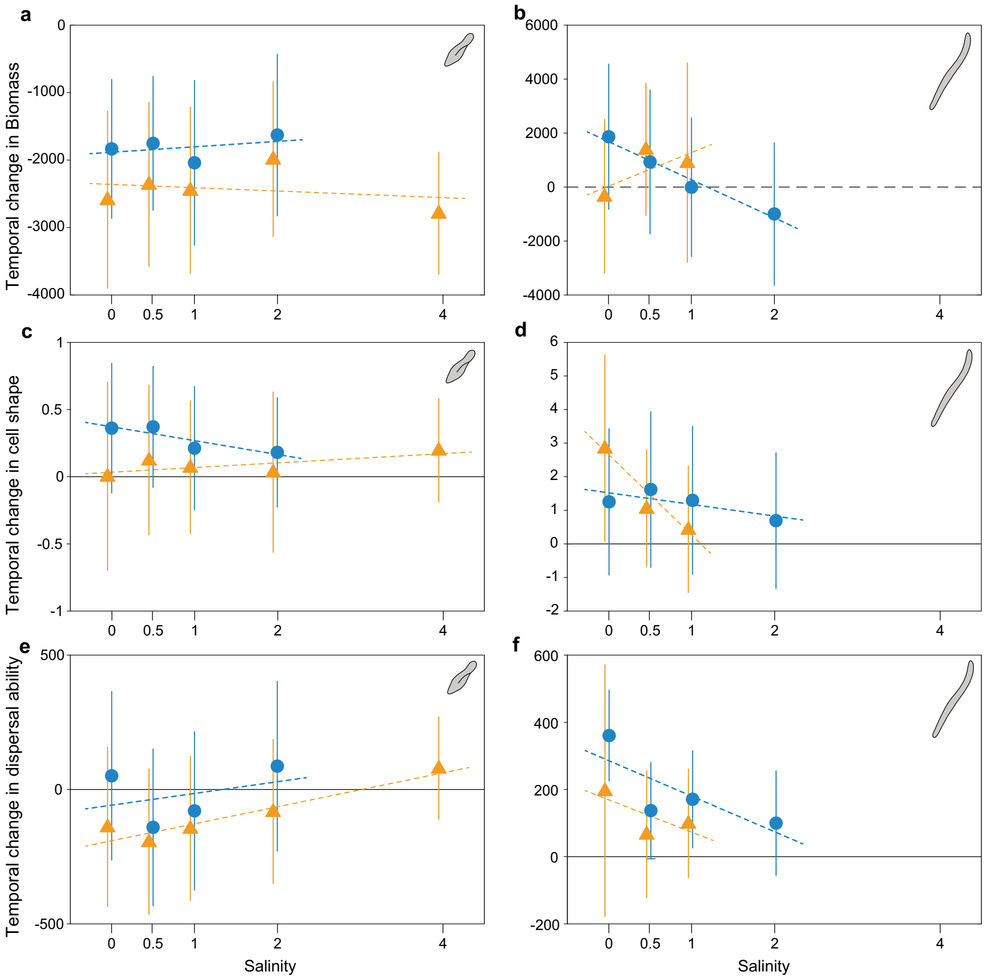

Figure S1: Temporal phenotypic difference during the selection phase (a-c) *Paramecium aurelia* and (d-f) *Spirostomum teres* along salinity and between populations in the absence and presence of competing species. Temporal phenotypic change for (**a**, **b**) biomass, (**c**, **d**) cell shape and (**e**, **f**) dispersal ability was calculated as the trait difference between all possible comparisons between individuals recorded at the start (day 4) and end (day 78) of the experimental evolution. Comparisons were drawn between individuals of the same microcosm. For representation purposes a unique mean across all microcosms is shown. Blue circles and yellow triangles represent mean values of the trait change in the absence and presence of competition, respectively. Error bars show standard deviations. Zero values given by the dashed line represent no change over time. Linear fits are a visual aid to see the trait change across salinity conditions. For most salinity conditions, *P. aurelia* responded to selection to the abiotic salinity environment by decreasing in biomass, becoming more elongated, and swimming slower. However, *P. aurelia* populations evolved with competing species decreased even more in bio-area, were less elongated, and swam even slower. Overall, *S. teres* individuals became larger, more elongated, and swam faster by the end of the evolution experiment. However, individuals evolved with competing species swam slower. The output of the statistical analysis on the effect of salinity and the presence of competition on trait change during the selection phase can be found in Supplementary Tables S1 and S2.

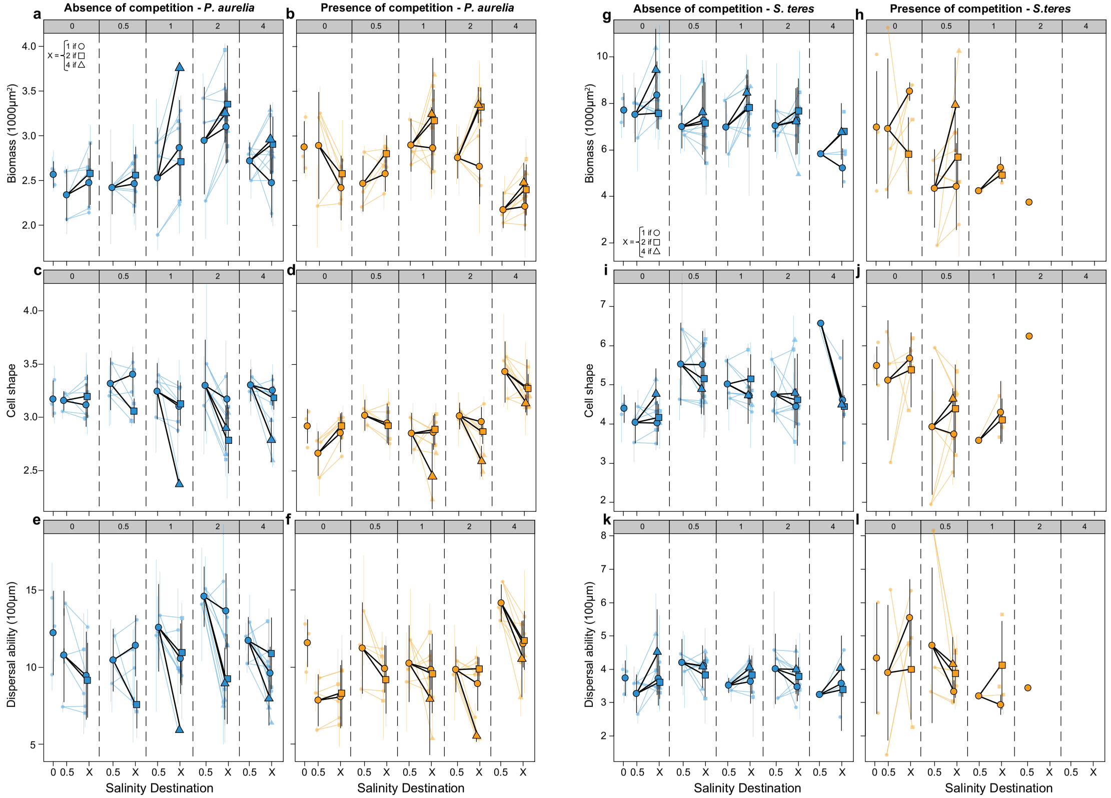

Figure S2: Common garden results of *Paramecium aurelia* and *Spirostomum teres* visualized as reaction norms in the absence and presence of competition. Trait values of **a-f**, *Paramecium aurelia* and **g-l**, *Spirostomum teres* for **a-b**, **g-h**, biomass (measured as mean bio-area; μm^2), **c-d**, **i-j** cell shape (measured as the ratio of the major to minor cell size axis), and **e-f**, **k-l** dispersal ability (measured as gross speed; μm) along the gradient of salinity conditions used in the selection experiment (different panels with grey headers) when evolved in the absence (blue colors) and presence (orange colors) of competition. Reaction norms are visualized by line segments connecting trait values between the 0.5 g/l salt condition (left circle) and the 1 g/l (right circle), 2 g/l (right square) and 4 g/l (right triangle), respectively. For the selected populations of the 0 g/l salt condition, trait values were also measured in the 0 g/l salt destination. Mean trait values across the three replicate microcosms are given by the large symbols. Mean trait values of each replicate microcosm is given by smaller transparent symbols. Error bars represent standard deviations around the mean.

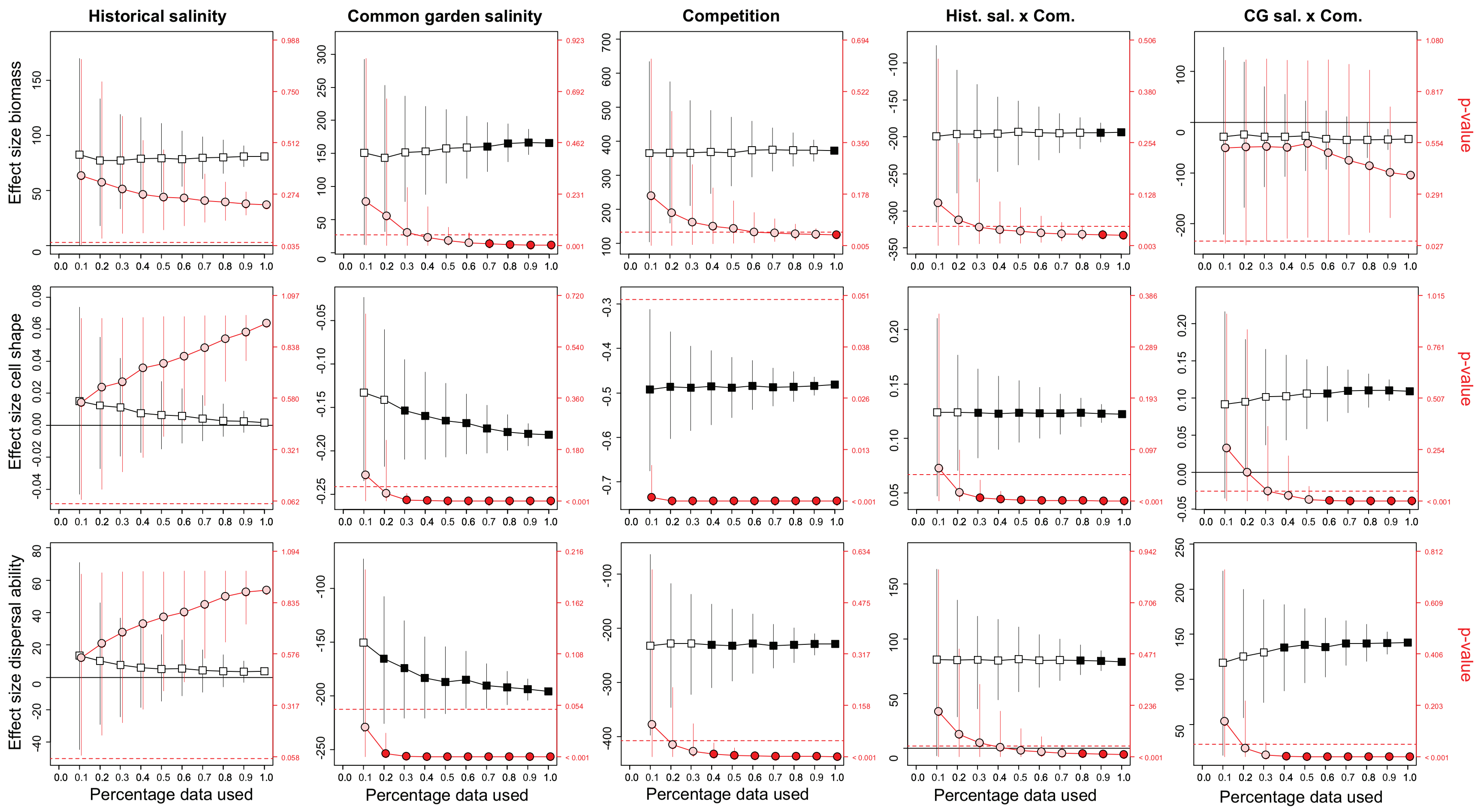

Figure S3: Robustness analysis for the effect sizes and their corresponding p-values obtained from regression analysis on the common garden data for *Paramecium aurelia*. For each effect size shown in Figure 3 in the main text, we here show the mean effect size (squares) obtained from 1000 bootstrap samples when bootstrapping the observed data along an interval of 10, 20, …, 90% of the original data. We also display the mean corresponding p-value (circles). Filled shapes indicate that the largest value of the 95% confidence interval of the bootstrapped p-values is smaller than 0.05.

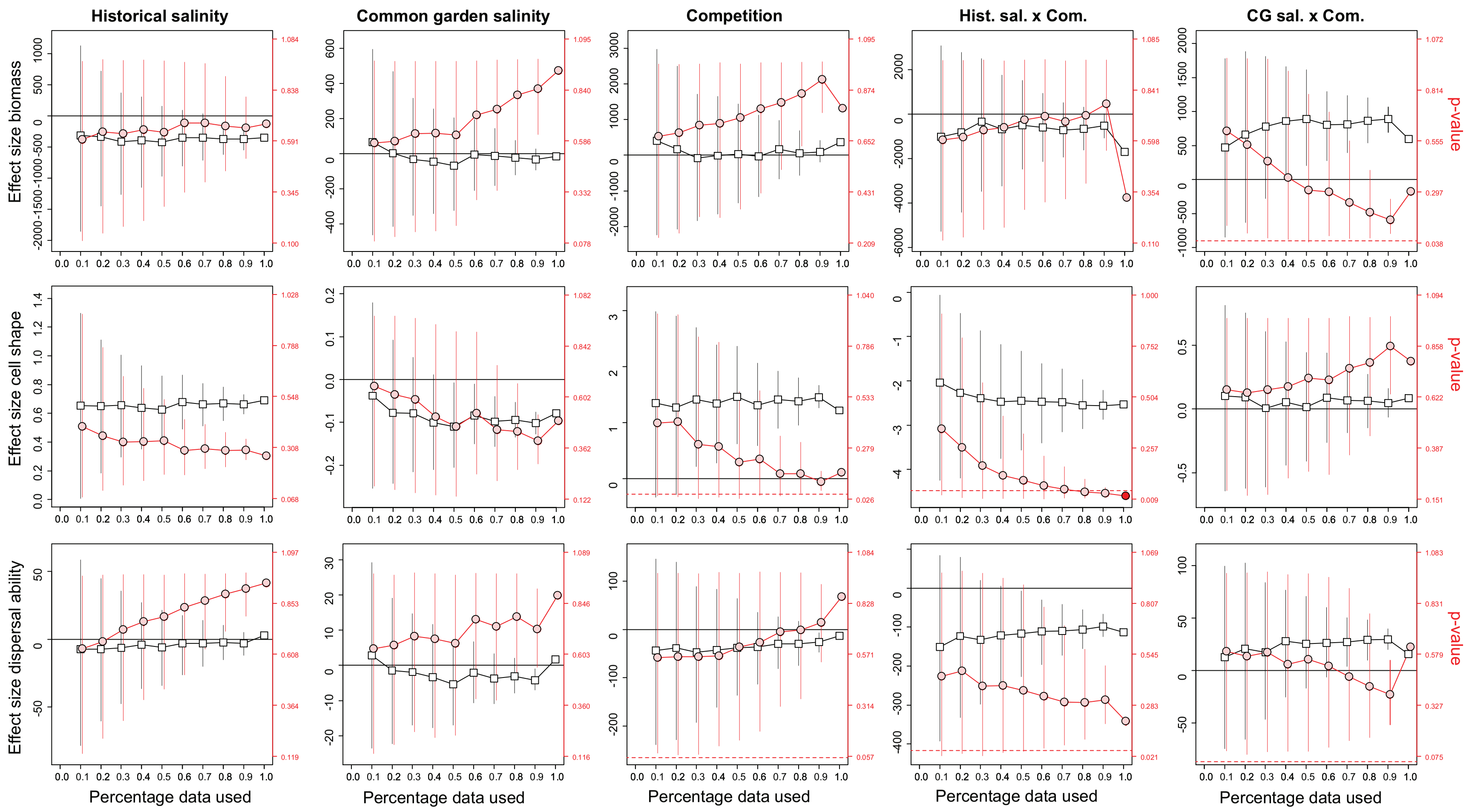

Figure S4: Robustness analysis for the effect sizes and their corresponding p-values obtained from regression analysis on the common garden data for *Spirostomum teres*. For each effect size shown in Figure 3 in the main text, we here show the mean effect size (squares) obtained from 1000 bootstrap samples when bootstrapping the observed data along an interval of 10, 20, …, 90% of the original data. We also display the mean corresponding p-value (circles). Filled shapes indicate that the largest value of the 95% confidence interval of the bootstrapped p-values is smaller than 0.05.

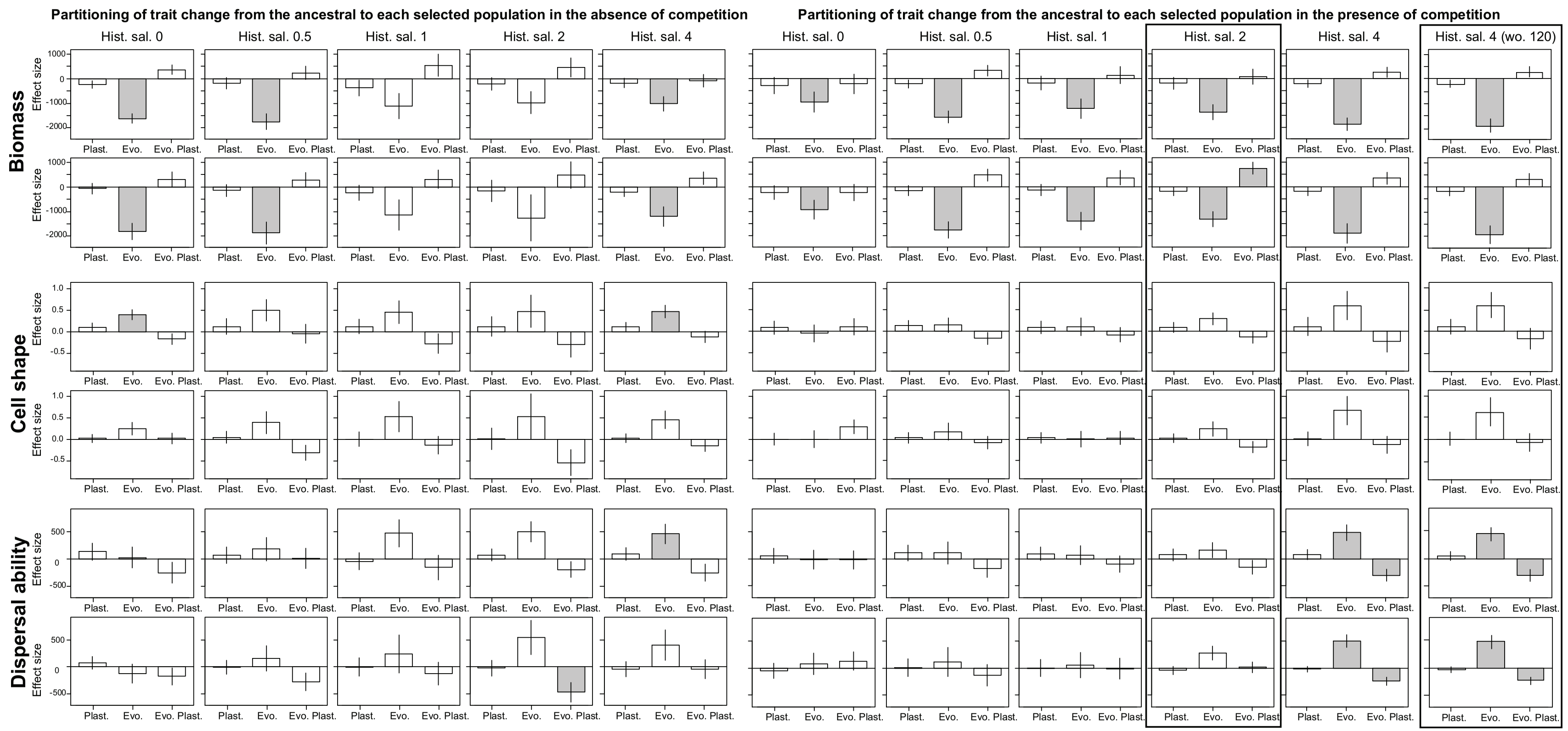

Figure S5: Reaction norm analysis for *Paramecium aurelia* quantifying trait change in biomass, cell shape and dispersal between the ancestral and each selected population. Partitioning of the observed trait change from the ancestral population at the start of the selection phase to each of the selected population measured in the common garden experiment. Effect sizes are obtained from a linear regression analysis for each of the components: (ancestral) plasticity (Plast.), mean trait evolution (Evo.) and evolution of plasticity (Evo. Plast.). Bars represent effect size, with error bars reflecting standard errors. Significant effects are given in grey. Solid rectangle around the two highest salinity conditions of the competition treatment reflect estimates of genetic trait change in the absence of *S. teres*, as *S. teres* went extinct in the highest salinity conditions (except in one replicate microcosm ID 120 for 4 g/l). Results with and without this replicate are displayed in the last two columns of the figure. Supplementary Tables S5-S6 show the detailed results of the statistical analysis.

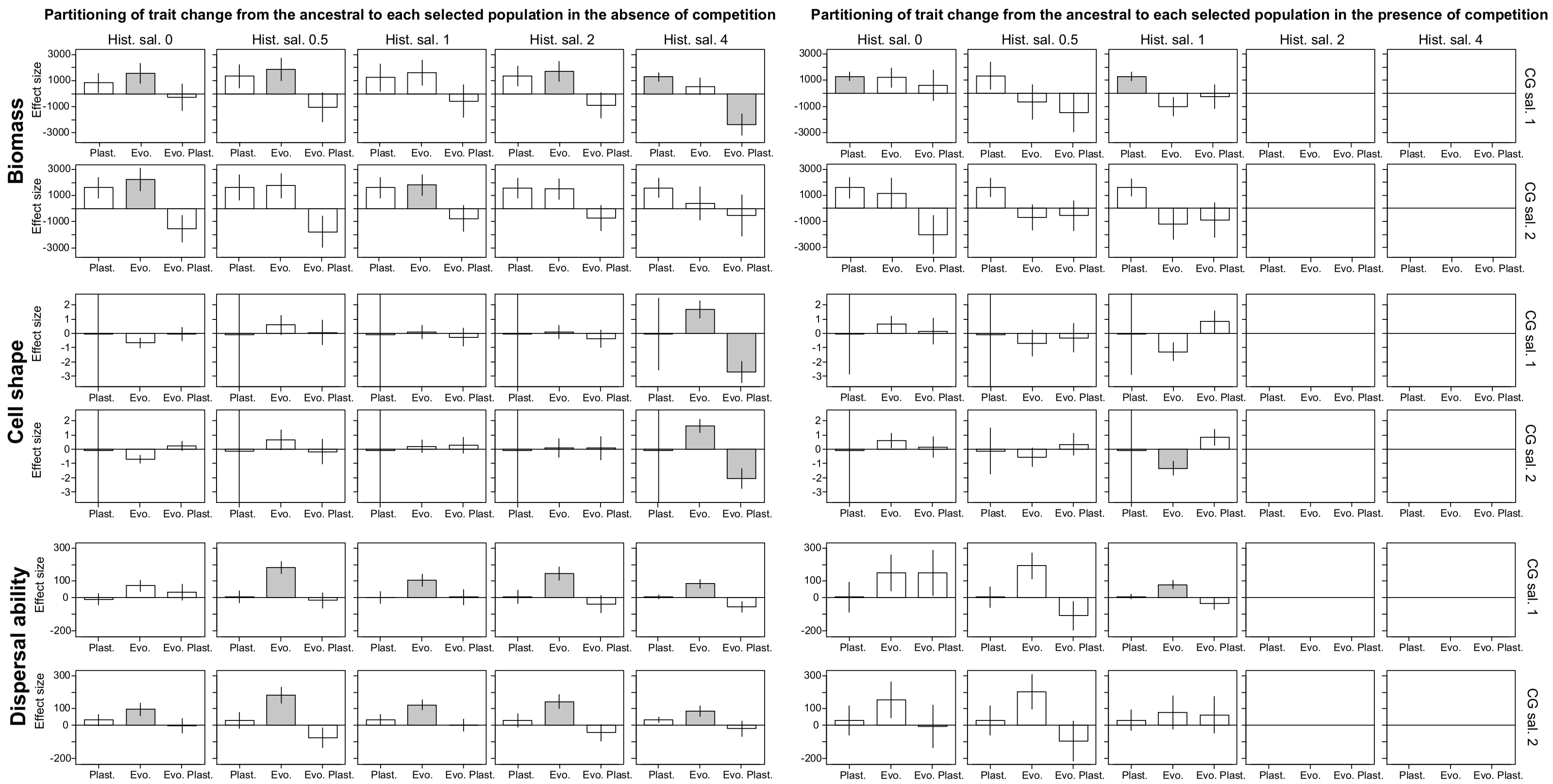

Figure S6: Reaction norm analysis for *Spirostomum teres* quantifying trait change in biomass, cell shape and dispersal between the ancestral and each selected population. Partitioning of the observed trait change from the ancestral population at the start of the selection phase to each of the selected population measured in the common garden experiment. Effect sizes obtained from linear regression analysis for each of the components: ancestral) plasticity (Plast.), mean trait evolution (Evo.) and evolution of plasticity (Evo. Plast.). Bars represent effect size, with error bars reflecting standard errors. Significant effects are given in grey. No partitioning could be done for the two highest salinity conditions of the competition treatment as *S. teres* went extinct in those conditions (except in one replicate microcosm ID 120 for 4 g/l; however only 1 individual was found, which was not enough to perform the reaction norm analysis using regression). Supplementary Tables S7-S8 show the detailed results of the statistical analysis.

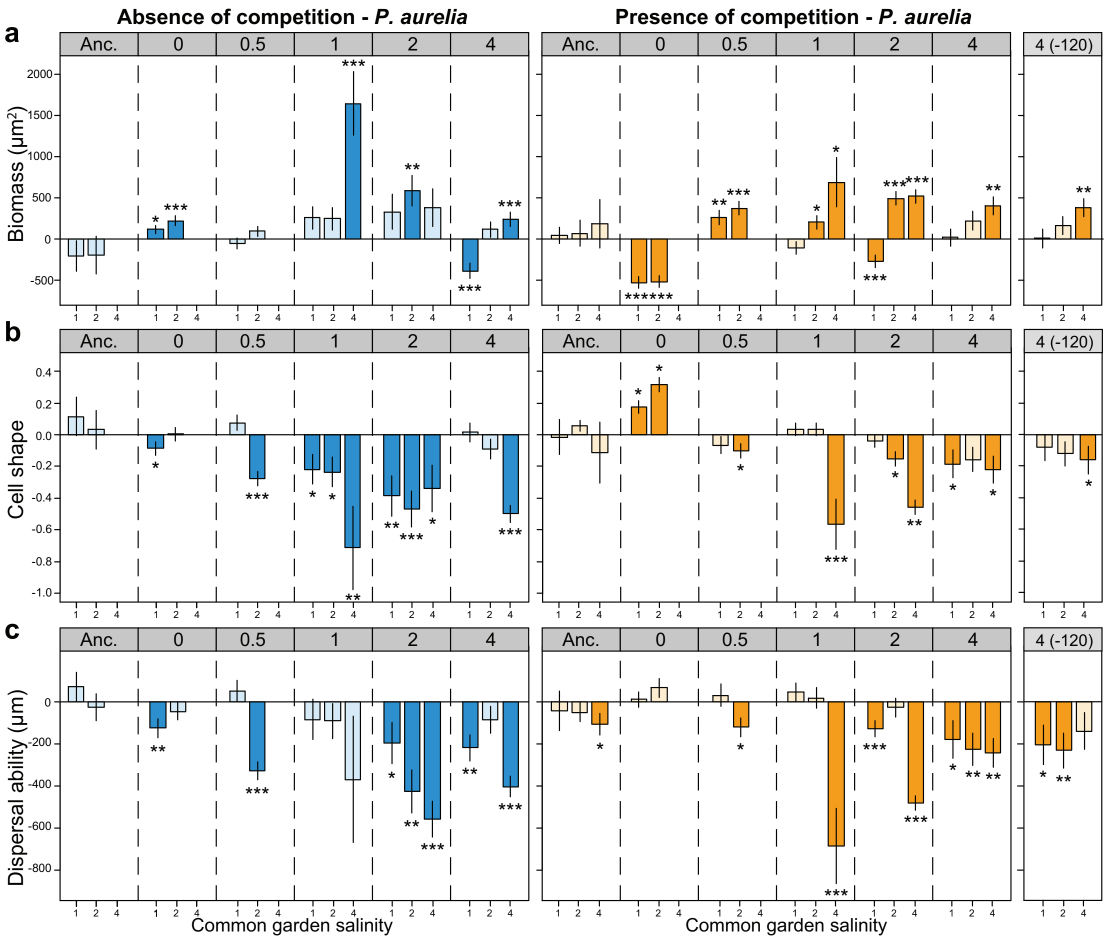

Figure S7: Phenotypic plasticity response to salinity of *Paramecium aurelia* for biomass, cell shape and dispersal ability of the ancestral (Anc.) and each of the selected (0, 0.5, 1, 2 and 4 g/l) populations evolved in the absence and presence of competitors. Bars display the magnitude (i.e. effect size) of the phenotypic plasticity responses to salinity and their standard errors of the ancestral (Anc.) (left panels) and each of the selected (0, 0.5, 1, 2 and 4 g/l) populations (remaining panels) for **a**, biomass (quantified as bio-area), **b**, cell shape (quantified as cell size ratio of the major and minor cell size axis) and **c**, dispersal ability (quantified as gross speed) for *P. aurelia* in the absence (blue) and presence (orange) of competition. Darker colors indicate significant effects, with asterisks referring to the level of significance; * < 0.05, ** < 0.01, *** < 0.001. The last column of the competition treatment displays the plasticity response to salinity when replicate microcosm ID 120 is excluded. Summary of statistical results can be found in Supporting Table S9-S10.

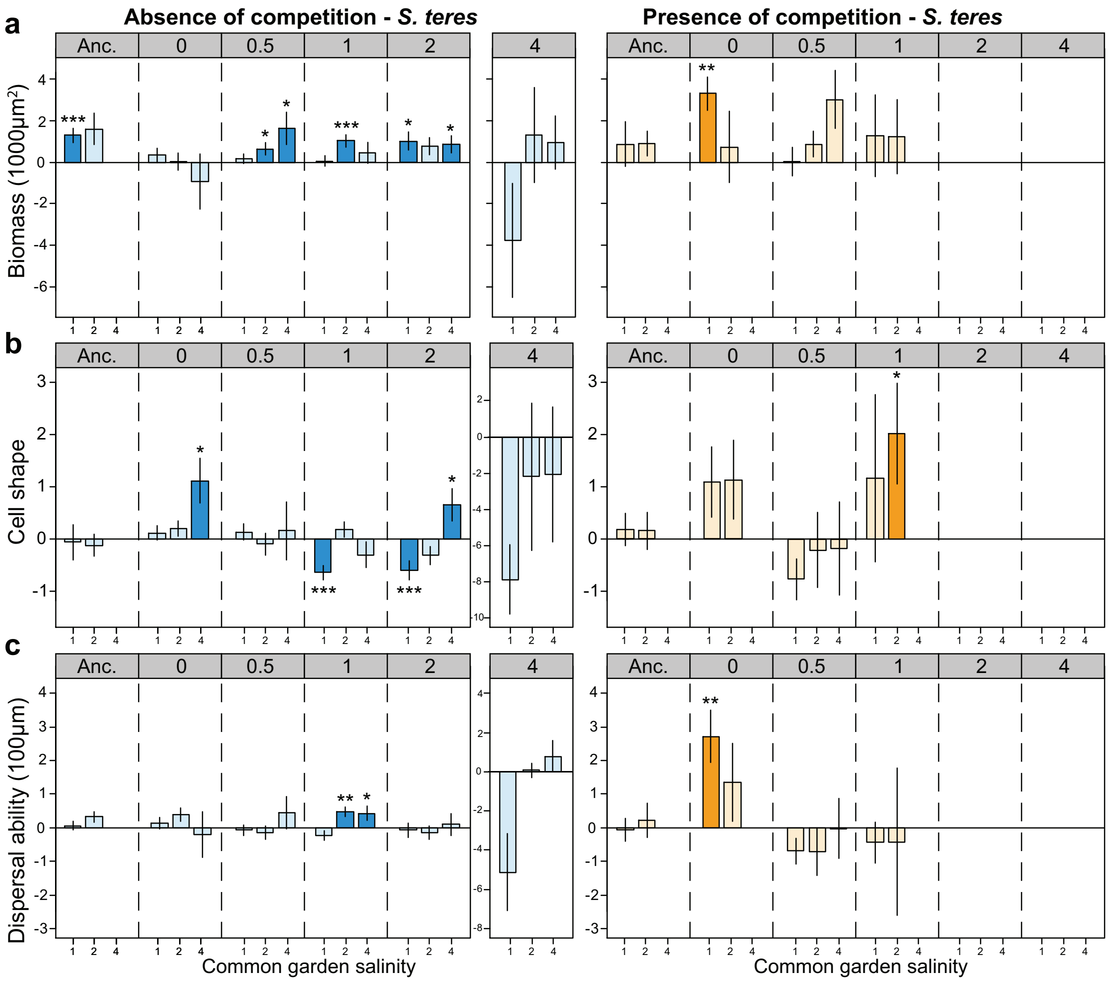

Figure S8: Phenotypic plasticity response to salinity of *Spirostomum teres* for biomass, cell shape and dispersal ability of the ancestral (Anc.) and each of the selected (0, 0.5, 1, 2 and 4 g/l) populations evolved in the absence and presence of competitors. Bars display the magnitude (i.e. effect size) of the phenotypic plasticity responses to salinity and their standard errors of the ancestral (Anc.) (left panels) and each of the selected (0, 0.5, 1, 2 and 4 g/l) populations (remaining panels) for **a**, biomass (quantified as bio-area), **b**, cell shape (quantified as cell size ratio of the major and minor cell size axis) and **c**, dispersal ability (quantified as gross speed) for *S. teres* in the absence (blue) and presence (orange) of competition. Darker colors indicate significant effects, with asterisks referring to the level of significance; * < 0.05, ** < 0.01, *** < 0.001. No plasticity response could be quantified for the two highest salinity conditions in the competition treatment due to extinction of the species. Summary of statistical results can be found in Supporting Table S11-S12.

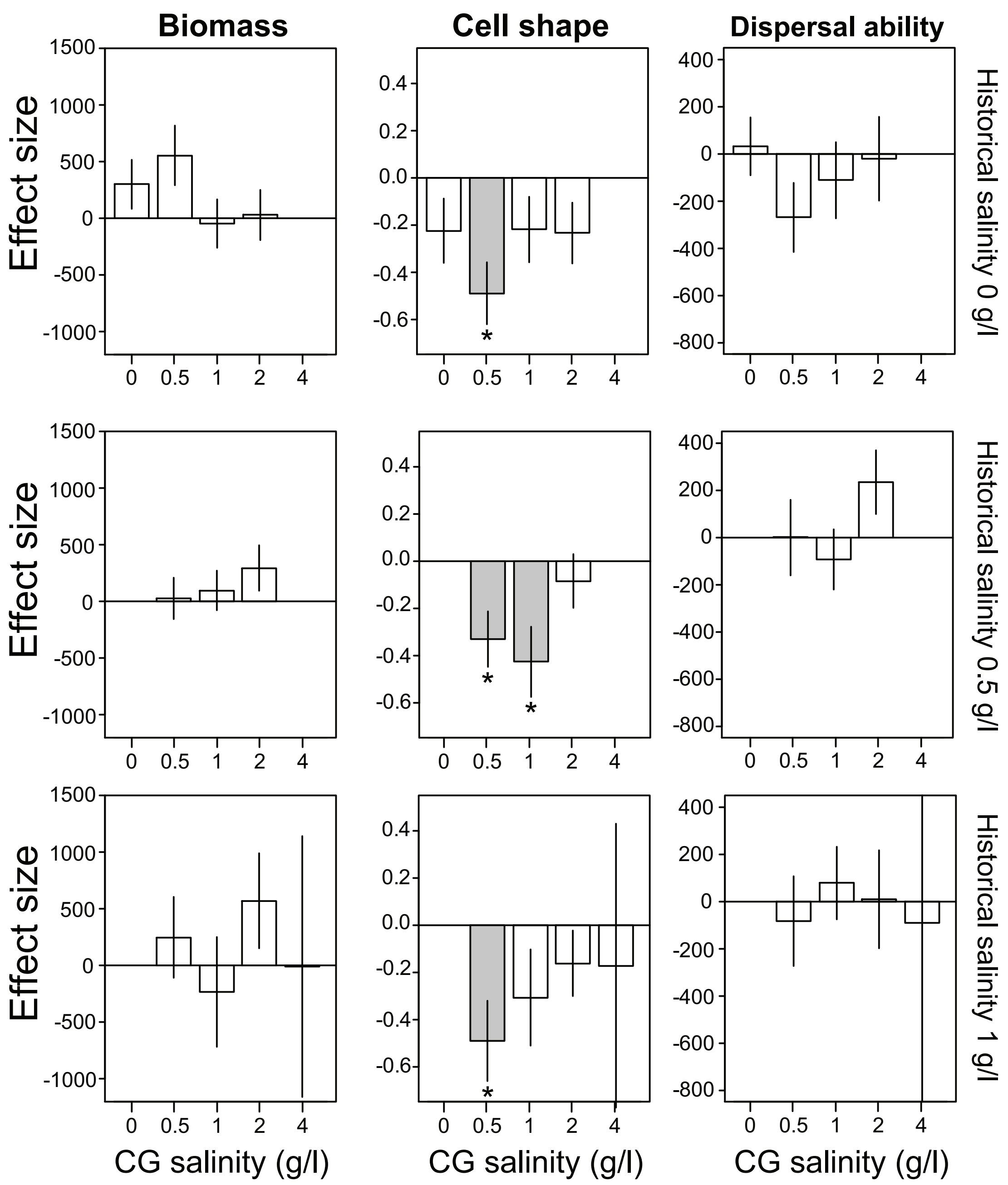

Figure S9: Trait difference due to historical competition and the presence of competitors for the salinity selected *Paramecium aurelia* populations evolved in the 0, 0.5 and 1 g/l salt conditions. Bars display the magnitude (i.e. effect size) of the trait difference due to historical competition and the presence of competitors and their standard errors for the selected populations evolved in the 0, 0.5 and 1 g/l salt conditions (given by salinity origin) for **a**, biomass (quantified as bio-area), **b**, cell shape (quantified as cell size ratio of the major and minor cell size axis) and **c**, dispersal ability (quantified as gross speed). Grey bars indicate significant effects, with asterisks referring to the level of significance; * < 0.05, ** < 0.01, *** < 0.001. Statistical results can be found in Table S14.

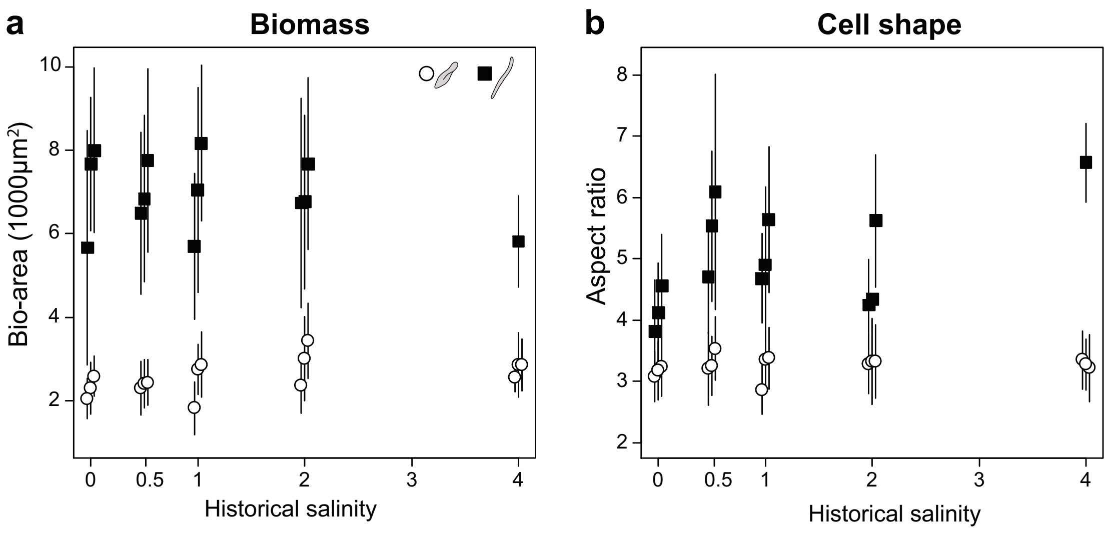

Figure S10: Biomass and cell shape values for *Paremecium aurelia* and *Spirostomum teres* obtained in the common garden of those populations evolved in the absence of competition along the salinity conditions used in the selection phase. **a**, Biomass (measured as bio-area, μm^2) and **b**, cell shape (measured as the cell size ratio of the largest to second largest cell size axis) for *P. aurelia* (unfilled circles) and *S. teres* (filled squares). Values reflect traits of the selected populations evolved in the different salt concentrations (0, 0.5, 1, 2 and 4 g/l) in the selection phase measured in the 0.5 g/l salt concentration common garden environment.

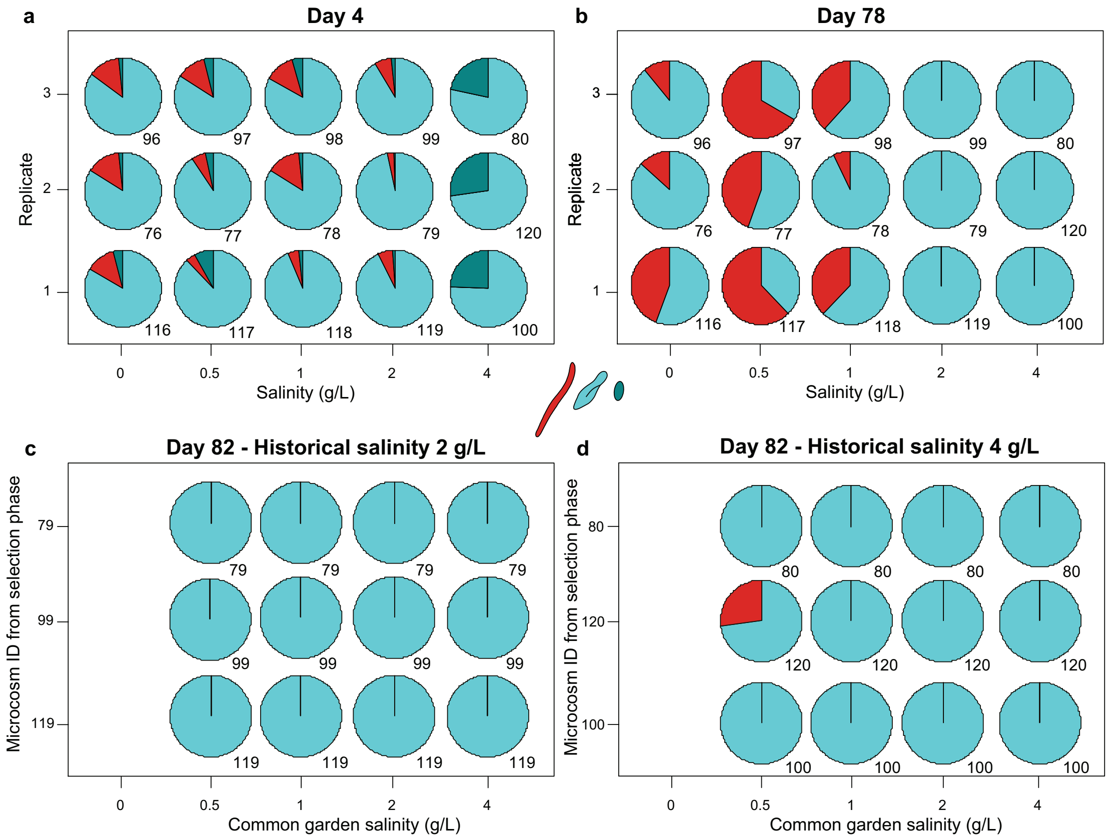

Figure S11: Community composition at the start (day 4) and end (day 78) of the experimental evolution and the common garden (day 82). Pie-charts represent relative species densities with respect to their biomass at a, start (day 4) and b, end (day 78) of the experimental evolution, and at the common garden (day 82) for c, historical salinity 2 g/l and d, historical 4 g/l.

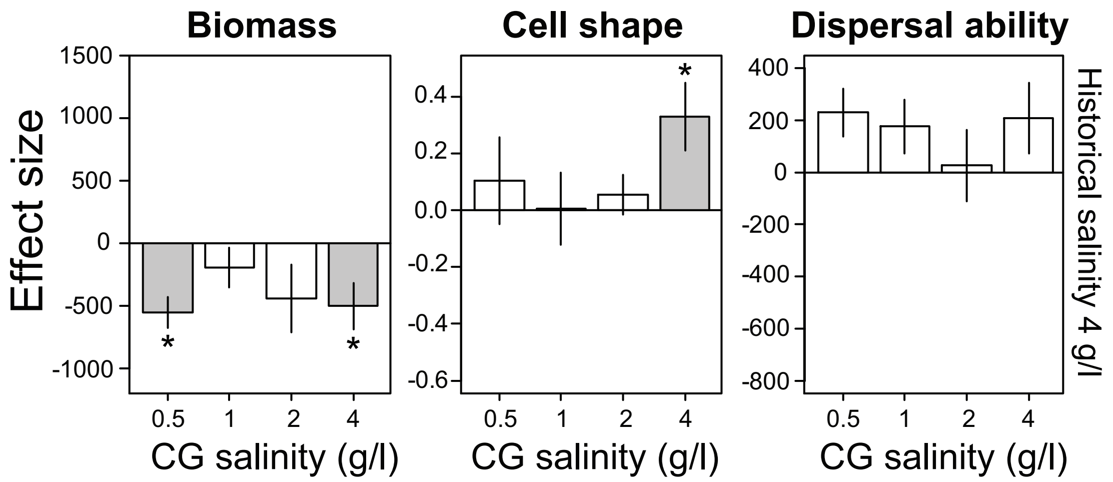

Figure S12: Genetic trait difference for the highest salinity selected *Paramecium aurelia* populations comparing those evolved in the absence and presence of competing species including microcosm ID 120. Bars display the magnitude (i.e. effect size) of the genetic trait difference along the different common garden (CG) salinity environments for the P. aurelia selected populations for biomass (quantified as bio-area), cell shape (quantified as cell size ratio of the major and minor cell axis) and dispersal ability (quantified as gross speed). Error bars reflect standard errors of the effect size as obtained from the regression model. Grey bars indicate significant effects, with asterisks referring to the level of significance; * < 0.05, ** < 0.01, *** < 0.001. Summary of statistical results can be found in Supplementary Table S13.
